# Polyphenolic Imidazopyridines as Multifunctional Modulators of Oxidative Stress, Metal Dyshomeostasis, and β_1-42_ Amyloid Aggregation in an In Vitro Model of Alzheimer’s Disease

**DOI:** 10.3390/antiox15070857

**Published:** 2026-07-08

**Authors:** Lidia Ciccone, Giovanni Petrarolo, Ilaria D’Agostino, Fabio Scianò, Bianca Laura Bernardoni, Manuela Leri, Jihyae Ann, Susanna Nencetti, Jeewoo Lee, Monica Bucciantini, Concettina La Motta

**Affiliations:** 1 Department of Pharmacy, University of Pisa, 56126 Pisa, Italy; 2 Department of Experimental and Clinical Biomedical Sciences “Mario Serio”, University of Florence, 50134 Florence, Italy; 3 College of Pharmacy, Seoul National University, Seoul 08826, Republic of Korea

**Keywords:** Alzheimer’s disease, Imidazo[1,2-*a*]pyridines, polyphenolic compounds, oxidative stress, metal chelation, Amyloid-β_1-42_ aggregation

## Abstract

Alzheimer’s disease (AD) involves oxidative stress, metal dyshomeostasis, and toxic oligomers of the amyloid-β peptide (Aβ_1-42_), calling for multifunctional agents. We investigated a panel of imidazo[1,2-*a*]pyridines bearing catechol or resorcinol motifs previously designed as SIRT1-activating agents. Their antioxidant profile was evaluated using in vitro DPPH and ABTS assays, which revealed promising radical scavenging activities, and TBARS assays on rat brain homogenates showing inhibition of lipid peroxidation, strictly dependent on the phenolic pattern. UV–Vis studies revealed metal-binding properties, particularly Cu^2+^ and Fe^2+^ interactions. In Aβ_1-42_ aggregation assays, the most active derivatives appeared to promote fibril maturation and the growth of large, ThT-low aggregates with distinct morphological features observed by TEM. Notably, Aβ_1-42_ aggregates generated in the presence of these compounds exhibited reduced cytotoxicity, preserved cell viability, and induced lower ROS levels in RA-differentiated SH-SY5Y cells compared to aggregates formed in their absence. Imaging and FRET analyses further indicated reduced formation of membrane-binding toxic species. Overall, our data suggest that polyphenolic imidazo[1,2-*a*]pyridines can remodel Aβ_1-42_ aggregation, redirecting it toward structurally distinct and less toxic assemblies, while also counteracting oxidative and metal-associated damage. These findings highlight their potential as multifunctional agents capable of addressing several pathological hallmarks of AD.

## 1. Introduction

Dementia is a growing global health concern, affecting over 55 million people worldwide in 2020, and this number is projected to rise dramatically, reaching 78 million by 2030 and 139 million by 2050 [1]. Approximately 10 million new cases are diagnosed each year globally, equivalent to one diagnosis every 3.2 seconds [2]. The economic impact is equally significant, with the annual global cost of dementia estimated at 1.3 trillion US dollars [3]. Collectively, these data highlight the growing burden of dementia as a major public health challenge.

Among the various forms of dementia, Alzheimer’s disease (AD) is the most prevalent one, accounting for 50% to 75% of all cases [4]. AD is a progressive neurodegenerative disorder characterized by memory impairment, cognitive decline, and behavioral disturbances. It significantly impacts patients’ quality of life, leading to a gradual deterioration of cognitive functions, associated with neuropil threads, selective neuronal loss, and synaptic degeneration [5].

The major challenge in treating AD lies in its pathogenesis: it is a multifactorial syndrome whose underlying mechanisms are not yet fully understood, involving a complex interplay of molecular, biochemical, and cellular processes that ultimately lead to neurodegeneration [6].

One of the earliest and most widely studied factors is the aggregation of β-amyloid peptide Aβ_1-42,_ which forms extracellular plaques that are highly toxic to neurons and disrupt synaptic integrity. Other recognized hallmarks are the reduced levels of acetylcholine, the key neurotransmitter for learning and memory functions, and the accumulation of hyperphosphorylated tau protein, which aggregates into flame-shaped neurofibrillary tangles (NFTs), destabilizing the cytoskeleton and impairing axonal transport [6].

Mitochondrial dysfunction and consequent oxidative stress are now considered central features of AD pathology. Impairment of mitochondrial function leads to excessive production of reactive oxygen species (ROS), resulting in oxidative damage to lipids, proteins, and nucleic acids, and overwhelming the endogenous antioxidant defenses [7]. Moreover, oxidative stress may also promote Aβ misfolding and aggregation, thereby contributing to a self-sustaining cycle of amyloid accumulation, mitochondrial dysfunction, and neuronal damage [8].

In addition, redox-active metal ion dyshomeostasis further exacerbates oxidative stress. Specifically, zinc contributes to Aβ_1-42_ aggregation and synaptic dysfunction and promotes tau pathology, while indirectly modulating oxidative stress pathways. Iron, which is tightly regulated in the healthy brain, contributes to both plaque and NFT formation and induces Aβ_1-42_ aggregation, whereas copper promotes Aβ_1-42_ production and enhances tau hyperphosphorylation [9].

Beyond these core mechanisms, Aβ_1-42_ accumulation may activate microglia and astrocytes, producing additional ROS [10] and inflammatory mediators that further contribute to neuronal dysfunction [11]. Vascular alterations and chronic cerebral hypoperfusion may additionally exacerbate Aβ_1-42_ and tau dysregulation, neuroinflammation, impairment of the blood–brain barrier (BBB) and mitochondrial function, and neuronal loss [12].

Although affecting a large portion of the worldwide population, no curative treatments for AD have been developed so far [13]. Current therapeutic approaches can be broadly classified into disease-modifying and symptomatic treatments. Disease-modifying treatments include monoclonal antibodies targeting Aβ_1-42_ aggregates, such as Lecanemab and Aducanumab, while the latter strategy comprises acetylcholinesterase inhibitors (e.g., Donepezil, Rivastigmine, Galantamine, historically Tacrine), as well as *N*-methyl-*D*-aspartate (NMDA) receptor antagonists, such as Memantine. Overall, these interventions alleviate cognitive symptoms but do not halt disease progression, underscoring the persistent unmet need for truly disease-modifying treatments [14].

Given the multifactorial nature of AD and the limited efficacy of currently available treatments, increasing attention has been directed toward molecular targets involved in multiple disease-related pathways. Among these, sirtuin 1 (SIRT1), a NAD^+^-dependent deacetylase, has emerged as a potentially relevant target in AD. Indeed, SIRT1 exerts multiple neuroprotective effects by promoting tau deacetylation, thereby limiting NFT formation, by enhancing α-secretase activity (e.g., ADAM-10), [15] reducing Aβ production, and upregulating SIRT6, which improves DNA repair and genomic stability [16]. Consistent with these effects, reduced SIRT1 levels and activity have been reported in the cortex of AD patients and are associated with the burden of Aβ plaques and NFTs [17].

In this context, therapeutic strategies increasingly aim to target multiple pathogenic pathways simultaneously, fostering interest in multifunctional agents capable of modulating more than one mechanism involved in AD progression [18,19,20,21,22,23,24]. Among these, phenolic compounds have attracted significant attention due to their pleiotropic properties, including antioxidant, anti-inflammatory, metal-chelating, and anti-amyloid activities [25,26,27,28,29].

**Figure 1:**
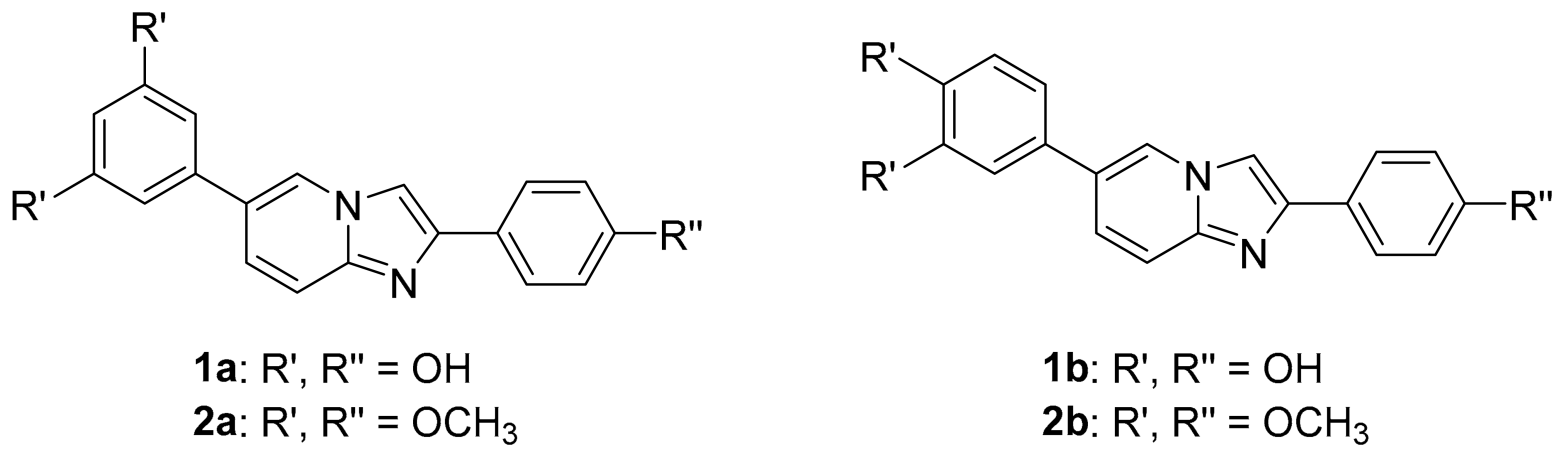
Structure of imidazo[1,2-*a*]pyridine compounds **1a-b** and **2a-b**.

In a previous work, an *in-house* library of polyphenolic imidazo[1,2-*a*]pyridine compounds, including compounds **1a-b** and **2a-b** (Figure 1), was investigated through an integrated in silico and in vitro screening platform for the identification of SIRT1 activators [30]. Specifically, molecular docking and MM-GBSA calculations predicted that all four derivatives could interact with the resveratrol-binding sites located within the *N*-terminal domain of SIRT1¸ while in the enzymatic assays, compound **1a** showed the strongest activation, reaching 79% of the effect elicited by resveratrol at 100 µM and displaying a dose-dependent profile. Compound **1b** also significantly activated SIRT1 activity, whereas the methoxy-substituted derivatives **2a** and **2b** yielded inconsistent and non-reproducible results.

Based on the involvement of SIRT1 in AD-related pathways and on our previous findings, the present exploratory study was designed to further investigate compounds **1a-b** and **2a-b** as potential multifunctional agents for future applicability in the treatment of AD. Specifically, the compounds were resynthesized through a revised and more straightforward synthetic pathway and subsequently evaluated for their antioxidant, metal-chelating, cytoprotective, and Aβ-modulating properties.

## 2. Materials and Methods

### 2.1. Synthesis

#### 2.1.1. General Chemistry

All commercially available chemicals and solvents were from Merck s.r.l. (Milan, Italy) and VWR Chemicals (Milan, Italy) and used as purchased. Analytical thin-layer chromatography (TLC) was carried out on Merck 0.2 mm precoated silica gel aluminium sheets (60 F-254), visualized under UV light. Chromatographic separations were carried out on silica gel (Merck) by manual flash chromatography or using an Isolera Prime automatic system (Biotage, Uppsala, Sweden). Evaporation was conducted under vacuum conditions in a rotating evaporator. Anhydrous sodium sulfate was used as a drying agent after extraction treatments. ^1^H NMR spectra were recorded on a Bruker Avance III HD 400 MHz spectrometer at 400 MHz and reported in parts per million (δ scale) and internally referenced to the DMSO-*d_6_* signal at δ 2.50 ppm. Data are shown as follows: chemical shift, multiplicity (s = singlet, d = doublet, t = triplet, dd = doublet of doublet, m = multiplet and/or multiple resonances), coupling constants (*J*) in Hertz (Hz), and integration. ^13^C-NMR spectra and melting points as reported by Flori et al. [30].

The purity of the test compounds was assessed by HPLC analysis [30]. Analyses were performed on a Shimadzu LC-20AD liquid chromatograph equipped with a photodiode-array (PDA) detector and a Luna C18 column (250 × 4.6 mm, 5 µm; Phenomenex). Different elution conditions were applied for the phenolic derivatives **1a** and **1b** and the methoxy derivatives **2a** and **2b**. All analyses were carried out at a flow rate of 1.0 mL/min over a 20 min run. For the phenolic derivatives, a gradient elution method was used with H_2_O containing 0.1% formic acid as solvent A and acetonitrile containing 0.1% formic acid as solvent B. The gradient was as follows: 5% B from 0 to 2 min, 5–20% B from 2 to 8 min, 20–35% B from 8 to 13 min, 35–60% B from 13 to 16 min, 60–95% B from 16 to 17 min, 95% B from 17 to 18.5 min, and 95–5% B from 18.5 to 19 min, followed by re-equilibration at 5% B until 20 min. Conversely, compounds **2a** and **2b** were analyzed under isocratic conditions using acetonitrile/H_2_O 70:30 *v*/*v*. Purity was determined by chromatographic peak-area normalization, and all tested compounds showed a purity ≥ 95%.

NMR characterization and HPLC chromatograms of the final compounds are reported in the Supplementary Material.

#### 2.1.2. Synthesis of Intermediates **3a-b**

In a reaction tube, 5-bromopyridin-2-amine (1 equiv.), the suitable phenylboronic acid (1.1 equiv.), and sodium carbonate (3 equiv.) were dissolved in degassed 5:1:1 ethanol/water/toluene (0.25 M) under a nitrogen atmosphere. Then, tetrakis(triphenylphosphine)palladium(0) (0.01 equiv.) was added, the tube was sealed, and the resulting mixture was stirred at 120 °C for 12 h. After cooling to room temperature, the reaction mixture was filtered under vacuum through a Celite^®^ pad using methanol, and the filtrate was evaporated under reduced pressure. The crude product was purified by flash column chromatography on silica gel using a petroleum ether/ethyl acetate (EtOAc) gradient, affording the corresponding 5-phenyl-2-aminopyridine intermediates **3a-b**.

**5-(3,5-dimethoxyphenyl)pyridin-2-amine (3a).** White solid. Yield: 97%. ^1^H NMR (400 MHz, DMSO-*d_6_*) δ: 8.25 (dd, *J* = 2.6, 0.8 Hz, 1H), 7.70 (dd, *J* = 8.6, 2.6 Hz, 1H), 6.69 (d, *J* = 2.2 Hz, 2H), 6.50 (dd, *J* = 8.8, 0.6 Hz, 1H), 6.40 (t, *J* = 2.2 Hz, 1H), 6.07 (s, 2H), 3.78 (s, 6H).

**5-(3,4-dimethoxyphenyl)pyridin-2-amine (3b).** White solid. Yield: 42%. ^1^H NMR (400 MHz, DMSO-*d_6_*) δ: 8.21 (dd, *J* = 2.6, 0.8 Hz, 1H), 7.66 (dd, *J* = 8.6, 2.6 Hz, 1H), 7.10 (d, *J* = 2.1 Hz, 1H), 7.05 (dd, *J* = 8.3, 2.1 Hz, 1H), 6.97 (d, *J* = 8.4 Hz, 1H), 6.49 (dd, *J* = 8.6, 0.8 Hz, 1H), 5.95 (s, 2H), 3.81 (s, 3H), 3.76 (s, 3H).

#### 2.1.3. Synthesis of Methoxy Derivatives **2a-b**

To a solution of the intermediate **3a-b** (1 equiv.) in absolute ethanol (0.13 M), sodium bicarbonate (2 equiv.) and 4-methoxybromoacetophenone (1.2 equiv.) were added. The resulting mixture was stirred under reflux for 3–5 h. After cooling to room temperature, the solvent was partially evaporated under reduced pressure, and the resulting solid was filtered and washed with water and petroleum ether. The crude products were then purified by flash column chromatography on silica gel (petroleum ether/EtOAc) to yield pure compounds **2a-b**.

**6-(3,5-dimethoxyphenyl)-2-(4-methoxyphenyl)imidazo[1,2-*a*]pyridine (2a).** Light brown solid. Yield: 41%. ^1^H NMR (400 MHz, DMSO-*d_6_*) δ: 8.89 (t, *J* = 1.5 Hz, 1H), 8.28 (s, 1H), 7.96–7.88 (m, 2H), 7.66–7.56 (m, 2H), 7.07–6.99 (m, 2H), 6.87 (d, *J* = 2.2 Hz, 2H), 6.54 (t, *J* = 2.2 Hz, 1H), 3.84 (s, 6H), 3.81 (s, 3H). The ^13^C NMR spectrum was consistent with that previously reported by Flori et al. [30]. HPLC-PDA purity = 98.9%, t_R_ = 6.55 min.

**6-(3,4-dimethoxyphenyl)-2-(4-methoxyphenyl)imidazo[1,2-*a*]pyridine (2b).** Pink solid. Yield: 51%. ^1^H NMR (400 MHz, DMSO-*d_6_*) δ: 8.82 (s, 1H), 8.27 (s, 1H), 7.91 (d, *J* = 8.3 Hz, 2H), 7.66–7.54 (m, 2H), 7.29 (s, 1H), 7.25 (d, *J* = 8.4 Hz, 1H), 7.07 (d, *J* = 8.3 Hz, 1H), 7.02 (d, *J* = 8.3 Hz, 2H), 3.87 (s, 3H), 3.81 (s, 6H). The ^13^C NMR spectrum was consistent with that previously reported by Flori et al. [30]. HPLC-PDA purity = 98.3%, t_R_ = 5.29 min.

#### 2.1.4. Synthesis of Hydroxy Derivatives **1a-b**

Derivative **2a-b** (1 equiv.) was dissolved in DCM (0.04 M) and cooled to −15 °C using an ice-salt bath. A commercially available solution of BBr_3_ (1.0 M in DCM, 6 equiv.) was added dropwise over 10 min. After stirring for an additional 10 min at −15 °C, the reaction mixture was allowed to warm to room temperature and stirred for 3 h. Upon completion, the reaction was quenched with aqueous NaOH (1 M, pH ≈ 8), and the mixture was extracted with DCM. The combined organic layers were dried over anhydrous Na_2_SO_4_, filtered, and concentrated under reduced pressure to afford the corresponding hydroxylated compounds **1a-b** as pure products.

**5-(2-(4-hydroxyphenyl)imidazo[1,2-*a*]pyridin-6-yl)benzene-1,3-diol (1a).** Light orange solid. Yield: 87%. ^1^H NMR (400 MHz, DMSO-*d_6_*) δ: 9.56 (s, 1H), 9.44 (s, 2H), 8.71 (d, *J* = 1.7 Hz, 1H), 8.22 (s, 1H), 7.88–7.69 (m, 2H), 7.57 (d, *J* = 9.3 Hz, 1H), 7.41 (dd, *J* = 9.3, 1.8 Hz, 1H), 6.92–6.75 (m, 2H), 6.51 (d, *J* = 2.1 Hz, 2H), 6.27 (t, *J* = 2.1 Hz, 1H). The ^13^C NMR spectrum was consistent with that previously reported by Flori et al. [30]. HPLC-PDA purity = 95.7% t_R_ = 15.08 min.

**4-(2-(4-hydroxyphenyl)imidazo[1,2-*a*]pyridin-6-yl)benzene-1,2-diol (1b).** Pinkish solid. Yield: 30%. ^1^H NMR (400 MHz, DMSO-*d_6_*) δ: 10.16 (s, 1H), 9.47 (s, 1H), 9.26 (s, 1H), 9.03 (s, 1H), 8.53 (s, 1H), 8.14–8.07 (m, 1H), 7.91 (d, *J* = 9.3 Hz, 1H), 7.84–7.75 (m, 2H), 7.14 (d, *J* = 2.3 Hz, 1H), 7.07 (dd, *J* = 8.2, 2.3 Hz, 1H), 7.03–6.93 (m, 2H), 6.92 (d, *J* = 8.2 Hz, 1H). The ^13^C NMR spectrum was consistent with that previously reported by Flori et al. [30]. HPLC-PDA purity = 98.0%, t_R_ = 15.27 min.

### 2.2. Antioxidant Activity Assays

#### 2.2.1. DPPH Assay

The DPPH assay was performed in a 96-well plate following a previously reported protocol [31]. Briefly, a freshly prepared DPPH solution in methanol (60 µM) was added to the tested compounds or to methanol as a control. After 1 h, in the dark box at room temperature, the plate was measured at 517 nm using a SPECTROstarNano UV-Vis spectrophotometer (220–1000 nm, Ortenberg, Germany). In the initial screening, all compounds were tested at a concentration of 100 µM. The percentage of radical scavenging activity (RSA%) was calculated using Equation (1):

(1) $$RSA\%=\frac{\left({Abs}_{DPPH}-{Abs}_{sample}\right)}{{Abs}_{DPPH}}\times 100$$

where *Abs_DPPH_* is the absorbance of the DPPH solution, and *Abs_sample_* is the absorbance of the DPPH solution containing the tested compound. For the most active compound (**1b**), the EC_50_ was determined. Each concentration was tested in triplicate, and results are reported in µM as best-fit estimates with the corresponding 95% confidence intervals (CI) in parentheses. Values were obtained by nonlinear regression analysis using the “log(inhibitor) vs. normalized response—Variable slope” model in GraphPad Prism 9; CI values were calculated by profile likelihood.

#### 2.2.2. ABTS Assay

The ABTS assay was carried out following previously reported protocols [32,33]. Briefly, 7 mM ABTS was mixed in a 1:1 ratio with 2.45 mM K_2_S_2_O_8_ in 10 mM phosphate buffer (pH 7.4). The day after, the resulting stock solution was diluted to an absorbance of 0.70 ± 0.02 at 734 nm. In a 96-well plate, the ABTS working solution was mixed with the samples **1a-b** and **2a-b** (100 µM), and absorbance was measured at 734 nm using a SPECTROstarNano UV-Vis spectrophotometer (220–1000 nm, Ortenberg, Germany). For the most active compound (**1b**), the EC_50_ was determined [34,35]. The EC_50_ values are reported µM as best-fit estimates with the corresponding 95% CI in parentheses. Values were obtained by nonlinear regression analysis using the “log(inhibitor) vs. normalized response—Variable slope” model in GraphPad Prism 9; CI values were calculated by profile likelihood.

#### 2.2.3. TBARS Assay

Male Wistar rats (12–15 weeks, 300–400 g) were housed under standard conditions (22 °C, 12 h light/dark cycle) with free access to food and water. All procedures complied with EU Directive 2010/63/EU and Italian Legislative Decree No. 26/2014 and were approved by the Animal Subjects Review Board of the University of Pisa (protocol no. 46/2023; codex DB173.N.IXS; 24 October 2023). Animals were euthanized under isoflurane anesthesia.

The TBARS assay was performed following a previously published protocol. Briefly, freshly isolated rat brains were homogenized in 10% (*w*/*v*) phosphate buffer (pH 7.4). Compounds **1a-b** and **2a-b** were screened at three different concentrations (1, 10, and 100 µM). The assay was carried out by treating rat brain homogenate with FeCl_3_ (20 µM) and ascorbic acid (100 µM), with or without the test compounds, and incubating at 37 °C for 30 min. After incubation, thiobarbituric acid (1% *w*/*v* in 0.05 N NaOH) and 25% *v*/*v* HCl were added, and the samples were boiled for 10 min. Then, the lipid peroxidation products were extracted with 3 mL of n-butanol, and the tubes were centrifuged. The organic layer was transferred to a 96-well plate, and absorbance was measured at 532 nm using a SPECTROstar Nano UV-Vis spectrophotometer (220–1000 nm, Ortenberg, Germany). TBARS levels were expressed as nmol of MDA per 10 mg of rat brain tissue, calculated from a 1,1,3,3-tetramethoxypropane standard curve. Data are reported as the mean ± SD of three independent measurements.

### 2.3. Metal Ion Chelation Assays

The selected compounds (**1a-b** and **2a-b**) were evaluated for their metal chelating properties using UV-Vis spectroscopy [36,37]. Absorbance spectra were recorded in the absence and presence of metal ions with a SPECTROstarNano UV-Vis spectrometer (220–1000 nm, Ortenberg, Germany). Experiments were carried out in 96-well plates, and each measurement was conducted in triplicate. The compounds (20 μM) in methanol were mixed with increasing concentrations of Cu^2+^ (CuCl_2_), Fe^2+^ (FeSO_4_), and Zn^2+^ (ZnCl_2_), ranging from 0 to 80 μM, and the solutions were incubated at room temperature for 30 min. The spectra were recorded, and absorbance changes were monitored over the 220–700 nm wavelength range. The stoichiometry of the ligand–metal complexes for Cu^2+^ and Fe^2+^ was determined using the mole ratio method [36].

### 2.4. Aβ_1-42_ Aggregation

The Aβ_1-42_ aggregation assay was performed following a previously published protocol [38]. Lyophilized Aβ_1-42_ peptide (Bachem, Bubendorf, Switzerland) was first dissolved in 100% hexafluoroisopropanol (HFIP) to a final concentration of 1.0 mM. The HFIP was allowed to evaporate overnight at room temperature, and the resulting peptide film was stored at −20 °C until further use. For aggregation studies, the peptide was re-dissolved in 20 mM sodium phosphate buffer (pH 7.4) to a final concentration of 25 μM and incubated at 25 °C. Samples were sonicated for 15 min and centrifuged at 18,000× *g* for 15 min at 4 °C. The concentration of soluble peptide in the supernatant was determined by UV absorbance at 280 nm using an extinction coefficient of ε_280_ = 1490 M^−1^·cm^−1^. Aggregation was monitored after 1 and 24 h at 25 °C without agitation, under conditions favoring the formation of oligomeric species. For co-aggregation experiments, Aβ_1-42_ (25 μM) was incubated with selected compounds at a peptide-to-compound molar ratio of 2.5:1. This aggregation protocol was used for TEM experiments and intrinsic fluorescence analyses as well as for all biological assays performed in this study. The DMSO-based protocol, described in Section 2.5, was not used for the biological assays because residual DMSO may affect cellular responses.

### 2.5. ThT Binding Assay

Aβ_1-42_ stock solution (250 μM) was prepared by completely dissolving the peptide in DMSO and diluting 10-fold with PBS to induce aggregation. This protocol was used exclusively for the ThT fluorescence measurements and was not employed for the biological experiments described above. Aggregation was measured by performing a ThT binding assay, in which the fluorescence intensity reflects the degree of Aβ_1-42_ fibril formation. Briefly, 5 μL of 250 μM Aβ_1-42_ and 45 μL of PBS were added to the well of the microplate. The compounds were added to the mixed solution at 10 μM concentration, and the mixtures were incubated for 1 h at r.t. ThT solution (150 μL of 5 μM ThT in 50 mM glycine, pH 8.5) was added to each well. The resulting ThT fluorescence was measured by using excitation and emission wavelengths of 440 and 485 nm, respectively.

### 2.6. Morphological Analysis

Aliquots (5.0 μL) of Aβ_1-42_ samples undergoing aggregation, with or without **1a** and **1b**, were collected at various time points. Each sample was applied to a Formvar/carbon-coated 400-mesh nickel grid (Agar Scientific, Stansted, UK), allowed to adsorb for 1–2 min, and then negatively stained with 2.0% phosphotungstic acid (PTA, Merck Italia s.r.l.) to enhance contrast for TEM imaging. Grids were air-dried and imaged using a JEM-1010 transmission electron microscope (JEOL Ltd., Tokyo, Japan) operated at an accelerating voltage of 80 kV.

### 2.7. Intrinsic Fluorescence

Intrinsic fluorescence spectra of Aβ_1-42_ were recorded using a BioTek Synergy H1 plate reader with an excitation wavelength of 275 nm. Emission was scanned from 300 to 430 nm. Measurements were performed on Aβ_1-42_ aggregates collected after 24 h of incubation, either alone or in the presence of **1a** or **1b**. Corresponding blank spectra, consisting of phosphate-buffered saline (PBS) alone or containing the respective compound at the same concentration used in the aggregation experiments, were acquired under identical conditions and subtracted from each sample to correct for background fluorescence contributions.

### 2.8. Cell-Based Assays

#### 2.8.1. Cell Culture and Differentiation of SH-SY5Y Cells

SH-SY5Y human neuroblastoma cells were maintained at 37 °C in a humidified atmosphere containing 5% CO_2_, in complete culture medium consisting of 50% HAM/F12 and 50% Dulbecco’s modified Eagle medium (DMEM), supplemented with 10% fetal bovine serum (FBS), 3.0 mM L-glutamine, 100 U/mL penicillin, and 100 μg/mL streptomycin (Merck Italia s.r.l.). Twenty-four hours after seeding, the serum concentration was reduced to 3.0%, and cells were treated with 10 μM all-trans retinoic acid (RA) to induce neuronal differentiation. Differentiation was carried out over five days, based on established protocols [39]. For MTT assays and ROS production, RA-differentiated SH-SY5Y (RA-SH-SY5Y) cells were seeded at a density of 1.5 × 10^4^ cells/well in 96-well plates. For immunofluorescence analysis, cells were plated at 5 × 10^4^ cells/well in 24-well plates.

#### 2.8.2. Cell Viability

Cell viability was evaluated using the 3-(4,5-dimethylthiazol-2-yl)-2,5-diphenyltetrazolium bromide (MTT) assay, optimized for RA-SH-SY5Y cells. Cells were then treated for 24 h with 2.5 μM Aβ_1-42_, aggregated for 1 and 24 h, in the presence or absence of **1a** and **1b** in 100 μL of serum-free DMEM (without phenol red) containing 0.5 mg/mL MTT. After incubation, 100 μL of lysis solution (100% DMSO) was added to each well, and the plate was incubated at 37 °C for an additional 2 h to ensure complete cell lysis. Absorbance of the resulting formazan product was measured at 595 nm using a BioTek Synergy H1 spectrophotometric plate reader. Final absorbance values were calculated by averaging triplicate wells for each condition and subtracting the blank (MTT solution + lysis solution without cells).

#### 2.8.3. Measurement of Intracellular ROS

Intracellular ROS levels were measured using the cell-permeant fluorescent probe 2′,7′-dichlorofluorescein diacetate (CM-H_2_DCFDA; Molecular Probes). This non-fluorescent compound is deacetylated by intracellular esterases and subsequently oxidized by ROS to form the highly fluorescent 2′,7′-dichlorofluorescein (DCF), which emits at 538 nm. Following 24 h of cell exposure to Aβ_1-42_ aggregates with or without **1a** and **1b**, 10 μM CM-H_2_DCFDA diluted in phenol red-free DMEM was added to the cells. After a 1 h incubation at 37 °C, fluorescence intensity was measured at 538 nm using a BioTek Synergy H1 plate reader.

#### 2.8.4. Immunofluorescence and FRET Analysis

Subconfluent RA-SH-SY5Y cells grown on glass coverslips were treated for 24 h with 2.5 μM Aβ_1-42_ aggregates formed in the presence or absence of **1a** and **1b**. After treatment, cells were washed with PBS and labeled for GM1 by incubation with 10 ng/mL Alexa Fluor 488-conjugated cholera toxin subunit B (CTX-B; Molecular Probes) in complete medium for 30 min at room temperature. Cell nuclei were stained with Hoechst 33342 for 30 min at 37 °C.

Cells were then fixed with 2.0% buffered paraformaldehyde for 10 min and permeabilized with a cold 1:1 acetone/ethanol solution for 4 min at room temperature. Following PBS washes, cells were blocked for 30 min with PBS containing 0.5% BSA and 0.2% gelatin. Aβ_1-42_ immunostaining was performed by incubating cells for 1 h at room temperature with a rabbit polyclonal anti-Aβ_1-42_ antibody (1:600 in blocking buffer), followed by a 30 min PBS wash under gentle stirring. Cells were then incubated with Alexa Fluor 568-conjugated anti-rabbit secondary antibody (Molecular Probes) diluted 1:200 in PBS for 1 h at room temperature. After staining, cells were washed twice with PBS and once with distilled water to remove non-specific binding.

Förster Resonance Energy Transfer (FRET) analysis was performed using the sensitized emission method according to Nosi et al. [40], where the sensitized emission signal is proportional to the ratio between acceptor fluorescence upon donor excitation (I_AD_) and acceptor fluorescence upon direct acceptor excitation (I_AA_) (2):

(2) $$E\propto \frac{I_{AD}}{\;I_{AA}}$$

where I_AD_ represents the acceptor emission detected following donor excitation and I_AA_ the acceptor emission following direct acceptor excitation.

FRET images were generated from donor, acceptor, and FRET channels after background subtraction and analyzed using Fiji/ImageJ software (version 1.54p) [41] to assess the close-range interaction between GM1-enriched membrane domains and cell-associated Aβ aggregates.

#### 2.8.5. Statistical Analysis

Statistical analyses were performed using OriginPro 2024 (OriginLab Corporation, Northampton, MA, USA). Data are presented as mean ± SE from at least three independent experiments. Comparisons between CTRL and Aβ-treated samples were performed using one-way analysis of variance (ANOVA) followed by Tukey’s multiple-comparison test. For cell-based assays involving Aβ aggregates generated under different aggregation conditions, the effects of aggregation time (1 h and 24 h), treatment (Aβ, Aβ + **1a**, and Aβ + **1b**), and their interaction were evaluated by two-way ANOVA followed by Tukey’s multiple-comparison test. The corresponding F-statistics, degrees of freedom, and *p*-values are reported in the figure legends and/or Section 3. Differences were considered statistically significant at *p* < 0.05.

## 3. Results

### 3.1. Synthesis of Compounds **1a-b** and **2a-b**

**Scheme 1:**
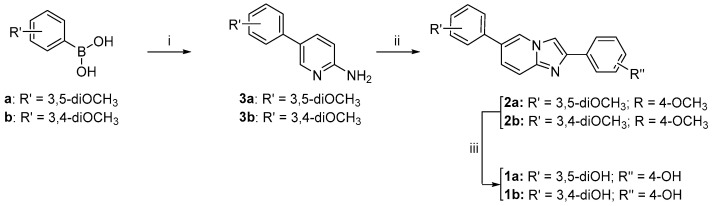
Synthesis of compounds **1a-b** and **2a-b**. Reagents and conditions: (**i**) 2-Amino-5-bromopyridine, Na_2_CO_3_, Pd(PPh_3_)_4_, Toluene/EtOH/H_2_O 5:1:1, sealed tube, 120 °C, 12 h, N_2_, 42–97%; (**ii**) Suitably substituted bromoacetophenone, NaHCO_3_, abs. EtOH, 80 °C, 3–5 h, 41–51%; (**iii**) BBr_3_, dry DCM, −15 °C, 10 min, then r.t., 3 h, 30–87%.

The synthesis of compounds **1a-b** and **2a-b** was performed as shown in Scheme 1, where a slight modification of a previously reported protocol [30] allowed a substantial reduction in reaction times and a marked increase in efficiency.

The commercial 2-amino-5-bromopyridine was first coupled with the suitably substituted phenylboronic acid under Suzuki–Miyaura cross-coupling conditions. Specifically, tetrakis(triphenylphosphine)palladium(0) was used as the catalyst in a toluene/EtOH/H_2_O (5:1:1) mixture under nitrogen in a sealed tube, at 120 °C for 12 h, affording the desired intermediates (**3a-b**) in excellent yields (42–97%). These intermediates were then reacted with the appropriate 4-methoxybromoacetophenone under mild basic conditions (sodium bicarbonate in refluxing absolute ethanol) for 3–5 h, yielding the corresponding 2-phenyl-imidazo[1,2-*a*]pyridines **2a-b**. Finally, selective demethylation was accomplished using boron tribromide in dichloromethane, at −15 °C for 10 min, followed by stirring at room temperature for 3 h, furnishing the corresponding hydroxy-substituted compounds **1a-b** in good yields.

### 3.2. Antioxidant Activity

To delineate the antioxidant profile of the synthesized compounds, **1a-b** and **2a-b**, two complementary assays, 2,2-diphenyl-1-picrylhydrazyl (DPPH) and 2,2′-azino-bis(3-ethylbenzothiazoline-6-sulfonic acid) (ABTS), were performed. These assays rely on colorimetric readouts, allowing an easy and rapid quantification of the antioxidant efficacy of compounds by monitoring changes in absorbance. Briefly, the DPPH assay measures the ability of test compounds to scavenge the stable free radical DPPH•, resulting in a decrease in its characteristic purple color, monitored at 517 nm. Similarly, the ABTS assay evaluates the capability of the test compounds to quench the ABTS•^+^ radical cation, causing a reduction in its blue-green color, observed at 734 nm. In both assays, the decrease in absorbance is proportional to the radical-scavenging activity (RSA) of the test compound.

In the DPPH assay, an initial screening of compounds **1a-b** and **2a-b** was performed at 100 µM, and the percentage of radical scavenging activity (RSA%) was determined (Table 1).

The RSA% values revealed pronounced differences in the antioxidant activity of the tested derivatives, depending on both the substitution pattern of the phenyl moiety (3.4 vs. 3.5) and the presence of methoxy (**2a-b**) or phenol (**1a-b**) functions. As expected, derivative **1b**, featuring the well-established radical scavenging catechol motif, showed the highest RSA% (=84). This could be attributed to the possibility of the catechol moiety forming a resonance-stabilized semiquinone intermediate. Concentration–response analysis confirmed its antioxidant potency, with an EC_50_ value of 49.2 µM. The resorcinol derivative **1a** proved to be less effective than the corresponding catechol analogue. Indeed, in this compound, the unpaired electron cannot be delocalized efficiently, so the resulting radical intermediate is less stable. Overall, methoxy derivatives showed lower or modest radical-scavenging activity compared with the catechol derivative **1b**, although **2a** showed a slightly higher RSA% than its phenolic counterpart **1a** in the DPPH assay.

The ABTS radical scavenging assay was performed on **1a-b** and **2a-b**, and the results are reported in Table 2.

As shown in Table 2, the phenolic derivatives **1a** and **1b** exhibited markedly higher antioxidant activity at 100 µM (RSA = 78–93%) than their methoxy-substituted counterparts **2b** and **2a**, which showed only modest radical scavenging effects (RSA values of 38% and 20%, respectively). In this assay as well, the antioxidant activity was found to strongly depend on the substitution pattern of the aromatic ring, with the catechol-containing derivative **1b** exhibiting the highest RSA value (93%), outperforming its positional isomer **1a** (78%). The EC_50_ value of compound **1b** was determined to be 13.7 µM; resveratrol was used as the reference compound.

**Figure 2:**
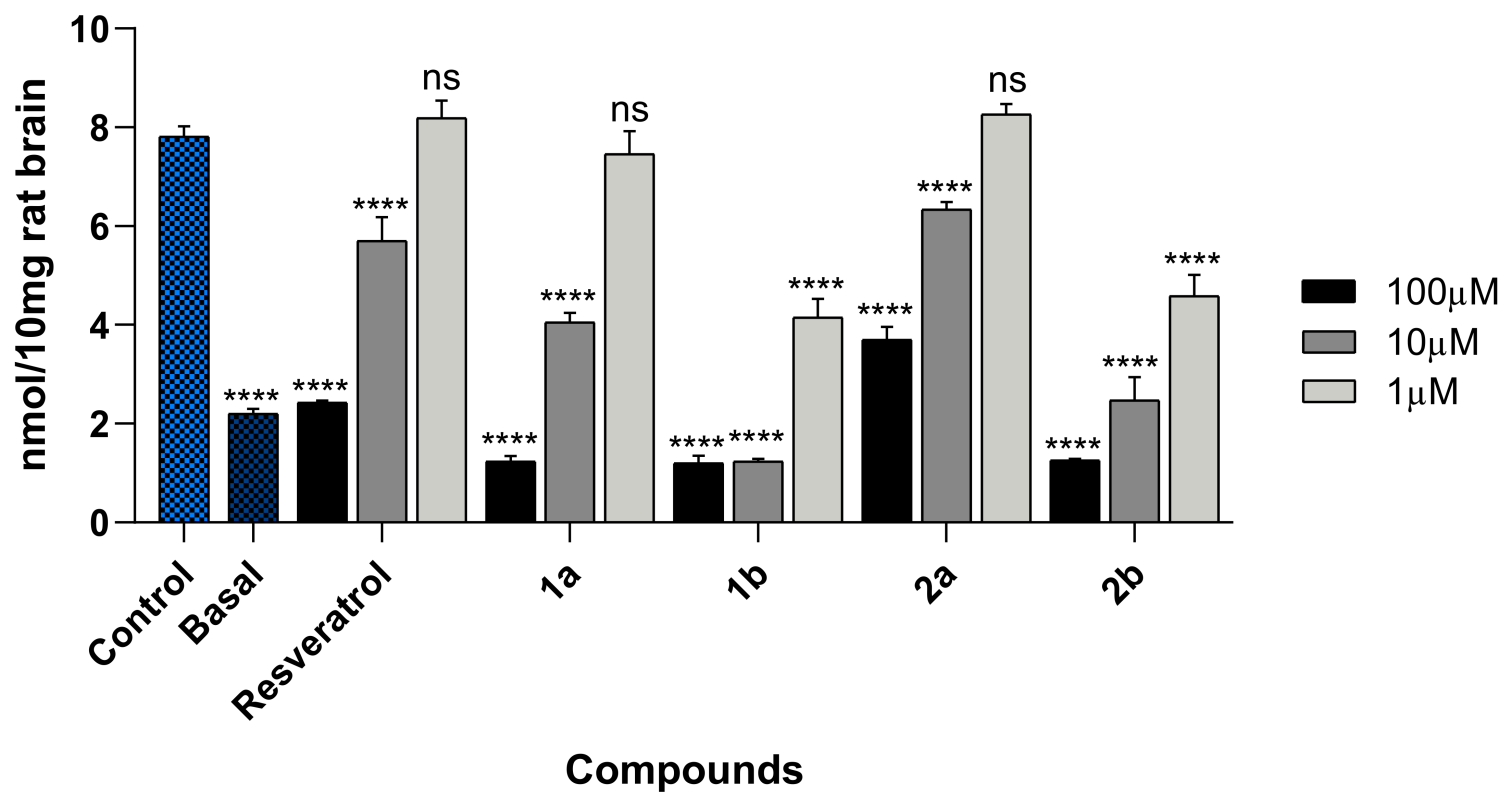
Effect of compounds **1a-b** and **2a-b** on the TBARS production in rat brain homogenates. The control and the basal conditions are shown in light blue and blue bars, respectively. Values are expressed as nmol of MDA equivalents, calculated from an MDA calibration curve. Lipid peroxidation was induced using the FeCl_3_/ascorbic acid system. Data represent the mean ± SD of three independent experiments. Statistical analysis was performed using one-way ANOVA followed by Tukey’s multiple-comparison test for comparisons with the control. **** *p* < 0.0001 vs. control; not significant (ns). All data are reported in Table S1.

Then, the compounds were evaluated in the thiobarbituric acid reactive substances (TBARS) test, carried out on rat brain homogenates treated with a traditional pro-oxidant system consisting of iron trichloride and ascorbic acid (Figure 2).

The test compounds and resveratrol, used as a reference antioxidant agent, were tested at three different concentrations (1, 10, and 100 µM), and the results were expressed as nmol of MDA per 10 mg of rat brain tissue. As shown in Figure 2, data obtained in the presence of the test compounds were compared with those of the control sample (brain tissue under pro-oxidant conditions), which showed the maximum detectable lipid peroxidation, and the basal brain condition (no treatment), which represents endogenous peroxidation. Oxidative induction caused a marked increase in MDA formation, and all the compounds reduced this rise to different extents (Figure 2 and Table S1). Specifically, the polyphenols **1a** and **1b** exhibited the strongest protective effect (84% and 85% of oxidation inhibition at 100 μM, respectively, Table S1), and apart from **2a**, all the compounds were more potent than resveratrol, reducing MDA levels below the basal condition when tested at 100 µM (69% oxidation inhibition, Table S1), and still exerting appreciable inhibition at 10 µM. In contrast, the 3,5-dimethoxy analogue **2a** showed weaker protection compared to resveratrol, even at 100 µM (53% oxidation inhibition, Table S1). These results indicate that free phenolic groups, particularly in a catechol arrangement, are critical for protection against lipid peroxidation, whereas *O*-methylation markedly attenuates this activity.

In summary, across the three assays performed, **1b** showed the strongest antioxidant efficacy thanks to its catechol motif, capable of effectively stabilizing radicals and resulting in promising ABTS radical-scavenging activity, and marked protection agaiNONpid peroxidation.

### 3.3. Metal Ion Chelating Activity

The metal-chelating ability of compounds **1a-b** and **2a-b** toward biometals Zn^2+^, Fe^2+^, and Cu^2+^ was evaluated by UV-Vis spectrophotometry [24,36]. In an initial screening, the spectra of the test compounds at 20 μM MeOH were recorded in the absence and in the presence of each metal ion at 80 μM, and then compared.

**Figure 3:**
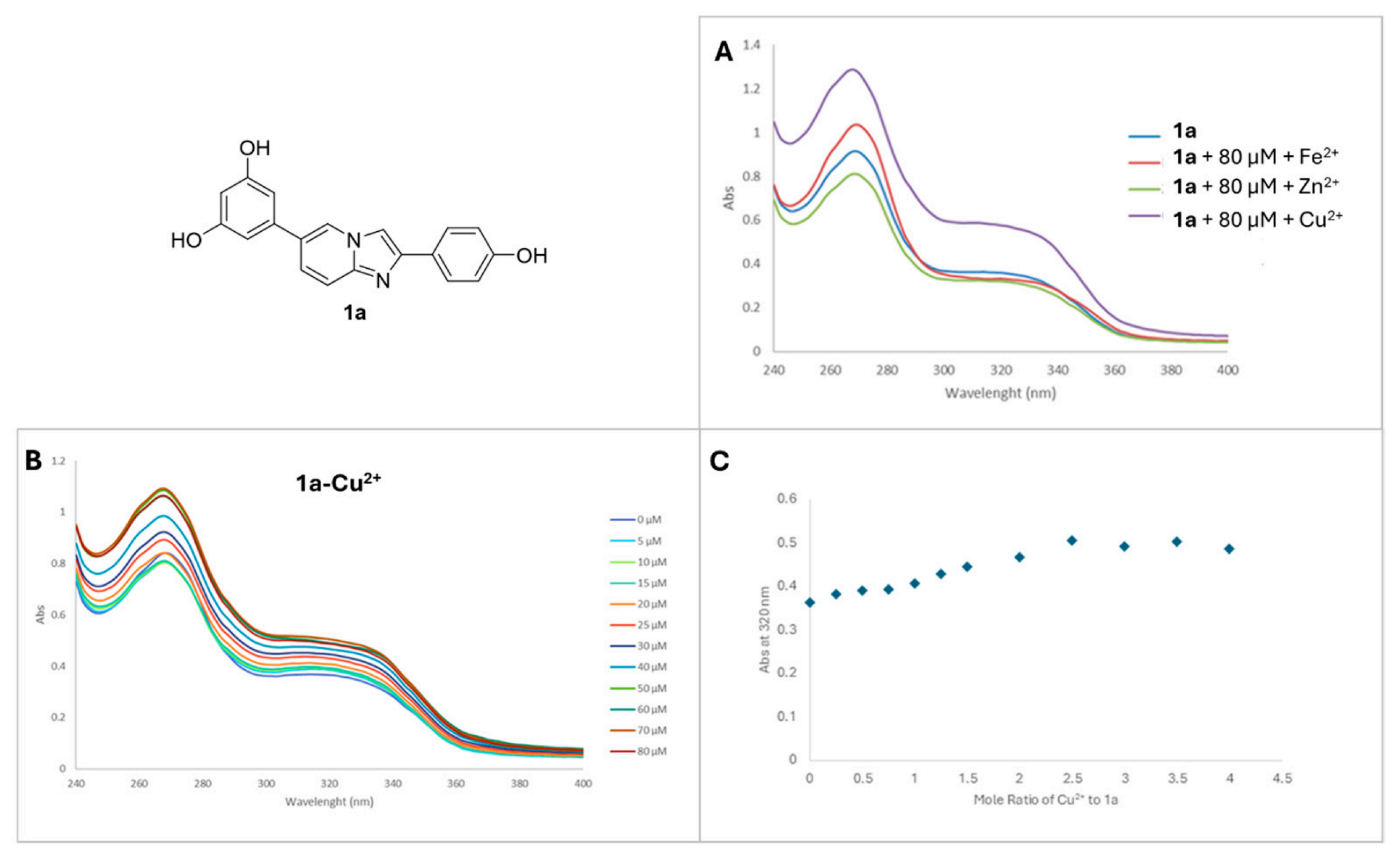
(**A**) UV spectra of **1a** (20 μM) in the presence of Zn^2+^, Cu^2+^, and Fe^2+^ (80 μM); (**B**) UV spectra of **1a** (20 μM) in the presence of different concentrations of Cu^2+^ (0–80 μM); (**C**) determination of the stoichiometry of the **1a**-Cu^2+^ complex.

Compounds **1a** and **2a** exhibited a clear alteration of their UV spectra upon addition of Cu^2+^; a representative spectrum from the screening is shown for derivative **1a** (Figure 3A).

For this compound, Cu^2+^ binding was evidenced by a shift in absorbance from 240 to 360 nm, accompanied by a pronounced hyperchromic effect, as indicated by the violet trace in Figure 3A. Following these preliminary observations, the stoichiometry of the Cu^2+^-**1a** complex was investigated using the molar ratio method [36]. A solution of **1a** (20 μM) was titrated with increasing concentrations of Cu^2+^ (0–80 μM), and the resulting absorbance changes were recorded (Figure 3B). Analysis of the molar fraction profile indicated a 2:1 stoichiometry for the Cu^2+^-**1a** complex, suggesting that one molecule of the resorcinol derivative coordinates two copper ions (Figure 3C).

Also, the UV spectra of catechol **1b** showed a shift in the absorption band from 250 to 350 nm in the presence of Fe^2+^, suggesting a 1:1 stoichiometry for the Fe^2+^-**1b** complex (Figure S1), whereas no appreciable spectral changes were observed for analogue **2b** with any of the tested metals (Figure S2). The 3,5-dimethoxy derivative **2a** exhibited a chelation profile similar to that observed for **1a**. In this case as well, the strongest interaction was observed with Cu^2+^, with a metal-to-ligand molar ratio of 2:1 (Figure S3).

Thus, considering the established role of Cu^2+^ and Fe^2+^ in promoting Aβ_1-42_ aggregation and ROS production in AD, the observed chelating properties may contribute synergistically to the overall neuroprotective profile of these compounds.

### 3.4. In Vitro Evaluation of Aβ_1-42_ Peptide Aggregation

#### 3.4.1. Thioflavin T Binding Assay

To evaluate the behavior of these compounds in the presence of the Aβ_1-42_ peptide, the Thioflavin T (ThT) binding assay was performed. This fluorescence-based method is widely used to monitor Aβ_1-42_ fibril formation [42]. Briefly, ThT selectively binds to β-sheet-rich structures within Aβ_1-42_ aggregates, leading to a marked increase in fluorescence intensity and a red shift in the emission maximum. Because the fluorescence intensity is proportional to the amount of fibrillar material formed, the assay enables a qualitative and quantitative assessment of Aβ_1-42_ aggregation.

**Figure 4:**
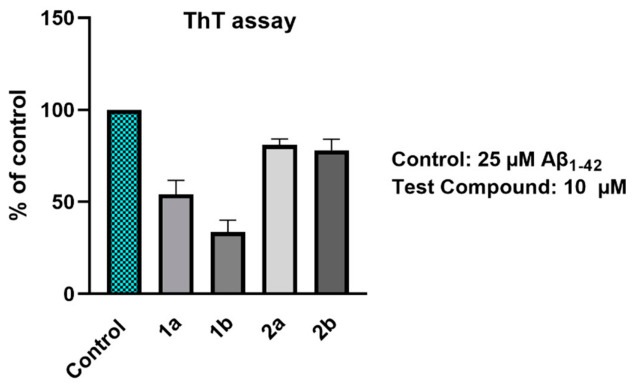
Thioflavin T (ThT) binding assay. Aggregation of Aβ_1-42_ into fibrils in the absence of compounds, used as control and represented by the light blue bar, or in the presence of compounds **1a-b** and **2a-b** at 10 µM. Data represent the mean ± SD of three independent experiments.The 3,5-methoxy derivative **2a** and its isomer **2b** produced only a moderate reduction in ThT fluorescence (80.9–78.0% of the control signal). Conversely, catechol derivative **1b** led to a much stronger decrease in ThT fluorescence (33.7%), whereas the resorcinol analogue **1a** produced a less pronounced, though still marked, reduction (54.1%).

Interestingly, the ThT assay results revealed that the tested compounds **1a-b** and **2a-b** were able to reduce ThT fluorescence compared with the control (100%, Aβ_1-42_ alone) when co-incubated with the Aβ_1-42_ peptide (Figure 4).

Importantly, a reduction in ThT fluorescence signal does not necessarily imply inhibition of fibril formation: ThT selectively binds to specific cross-β structural arrangements, and its fluorescence depends on the accessibility and organization of β-sheet binding grooves. Therefore, the observed decrease in fluorescence may reflect two, not mutually exclusive, scenarios: the formation of structurally distinct, ThT-low fibrillar species or higher-order, non-fibrillar aggregates with reduced dye affinity, rather than a true suppression of fibrillogenesis. Therefore, the compounds may either interfere with the formation of canonical ThT-positive fibrils or redirect Aβ_1-42_ assembly toward alternative, ThT-low aggregated species.

#### 3.4.2. Microscopy Observations

**Figure 5:**
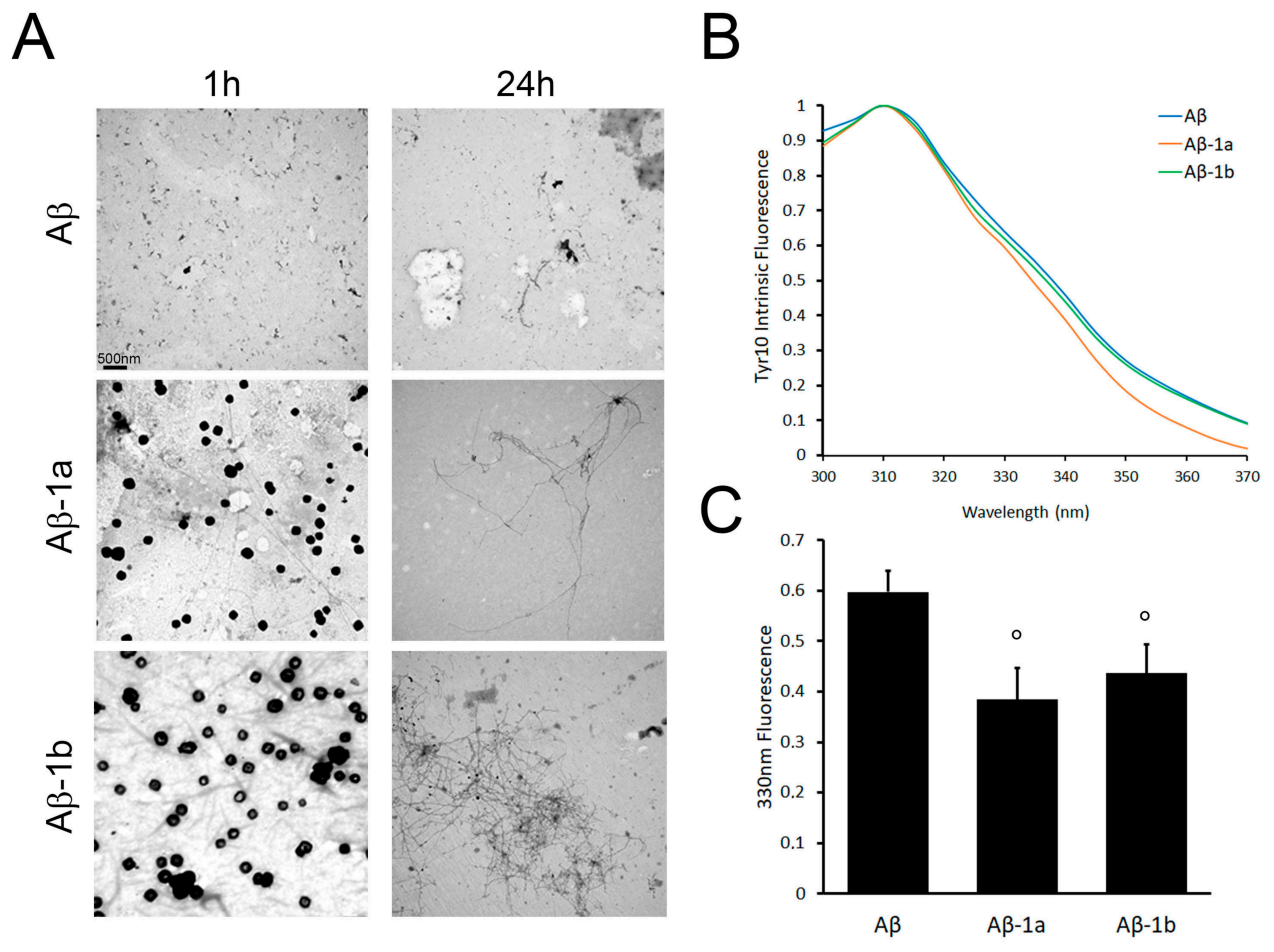
Compounds **1a** and **1b** promote Aβ_1-42_ fibrillogenesis and induce structural rearrangements. (**A**) TEM analysis of Aβ_1-42_ aggregates formed after 1 and 24 h of incubation, either alone (Aꞵ) or in the presence of compounds **1a** and **1b** (Aꞵ-**1a**, Aꞵ-**1b**); scale bars: 500 nm; (**B**) Intrinsic tyrosine fluorescence spectra of Aβ_1-42_ aggregates under the same conditions; (**C**) Quantification of the fluorescence emission at the 330 nm peak corresponding to tyrosine (Tyr10) fluorescence emission. Data are presented as mean ± SE from three independent experiments. Statistical analysis was performed using one-way ANOVA followed by Tukey’s multiple-comparison test. ° *p* < 0.05 vs. Aβ.

To investigate the effects of compounds **1a** and **1b** on the aggregation of Aβ_1-42_ peptides, transmission electron microscopy (TEM) and intrinsic tyrosine fluorescence assays were employed. TEM analysis revealed that both compounds appeared to accelerate Aβ_1-42_ fibrillogenesis, as demonstrated by the increased abundance and density of fibrillar structures following 1 h and 24 h of incubation relative to Aβ_1-42_ alone (Figure 5A). Notably, samples incubated with either compound displayed substantial accumulation of mature fibrils as early as 1 h, with this effect becoming more pronounced at 24 h, indicative of a pro-aggregating activity.

Moreover, TEM micrographs revealed the presence of spherical and vesicular aggregate structures. Their exact nature cannot be established from negative-stain TEM images alone, as sample preparation and drying procedures may contribute to the observed appearance. Further biophysical investigations performed under aqueous conditions will be required to determine whether these structures represent specific aggregation intermediates or arise during sample preparation. To assess conformational changes induced by these compounds, intrinsic tyrosine fluorescence emission, primarily arising from Tyr10, was measured. A significant decrease in tyrosine fluorescence intensity at 330 nm was observed in samples treated with compounds **1a** and **1b** compared with untreated Aβ_1-42_ (Figure 5B,C). Since the spectra were corrected by subtraction of the corresponding compound blanks, this fluorescence change is unlikely to arise from direct spectral contributions of the compounds. Rather, it is consistent with alterations in the local environment of Tyr10 accompanying the aggregation process. When considered together with the TEM observations and ThT measurements, these findings support the conclusion that compounds **1a** and **1b** modify the aggregation pathway of Aβ_1-42_, promoting the formation of structurally distinct fibrillar assemblies. Based on these results, compounds **1a** and **1b** were selected for further cell-based investigations.

### 3.5. Cell-Based Investigation

#### 3.5.1. Cellular Model for AD Assays

To evaluate the biological effects of the polyphenolic compounds in a more physiologically relevant context, we used the human neuroblastoma-derived SH-SY5Y cell line as a neuronal model. In their undifferentiated state, SH-SY5Y cells are immortalized, immature catecholaminergic-like cells that display neurite-like processes and express early neuronal markers. Because AD primarily affects cholinergic neurons, differentiation of SH-SY5Y cells is commonly employed to achieve a more neuron-like phenotype. Differentiation was induced by treatment with retinoic acid (RA), leading to RA-differentiated SH-SY5Y (RA-SH-SY5Y) cells characterized by enhanced neuronal features and upregulation of neuronal markers. Although these cells do not fully recapitulate the electrophysiological properties of mature neurons, they exhibit elongated neurites, increased expression of differentiation markers, and reduced proliferation rates, resembling key morphological aspects of human neurons. Overall, RA-SH-SY5Y cells provide a more physiologically relevant neuronal context than undifferentiated cells, supporting their use in cell-based evaluation of neuroprotective effects.

#### 3.5.2. Biological Properties of Aβ_1-42_ Aggregates Formed in the Presence of **1a** and **1b**

**Figure 6:**
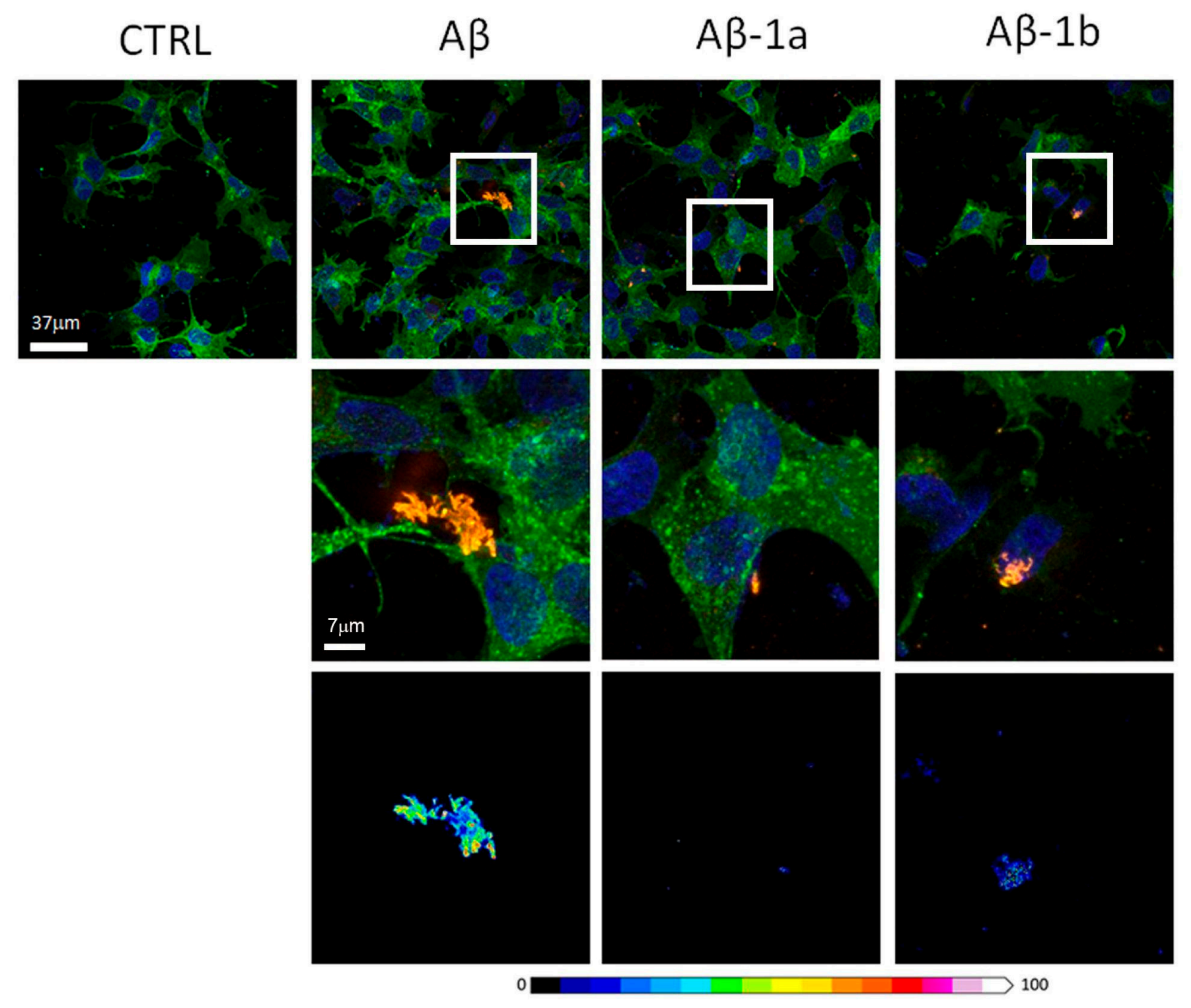
Reduced membrane binding of Aβ_1-42_ aggregates generated in the presence of compounds **1a** and **1b.** Representative confocal microscopy images of RA-SH-SY5Y cells exposed for 24 h to Aβ_1-42_ aggregates formed either in the absence (Aβ) or in the presence of compounds **1a** and **1b** (Aβ-**1a**, Aβ-**1b**). Nuclei are stained with Hoechst 33342 (blue), plasma membranes are labeled with the GM1 marker (green), and Aβ_1-42_ aggregates are visualized using Alexa Fluor 568-conjugated anti-Aβ antibodies (red). Top row: overview images (scale bar: 37 μm). Middle row: magnified regions corresponding to the areas highlighted by white squares in the top row, showing the distribution and membrane association of Aβ aggregates (scale bar: 7 μm). Bottom row: sensitized-emission FRET images showing the interaction between GM1-enriched membrane domains (donor, Alexa Fluor 488) and cell-associated Aβ aggregates (acceptor, Alexa Fluor 568). An increased FRET signal indicates close-range donor–acceptor interactions. Images are representative of five independent experiments.

To evaluate the biological relevance of Aβ_1-42_ aggregates formed under our experimental conditions, we investigated their interaction with cellular membranes, particularly GM1-enriched domains, which serve as key binding sites for toxic amyloid species and mediate amyloid-induced cellular dysfunction [43,44]. Despite promoting Aβ_1-42_ fibril formation, the aggregates generated in the presence of compounds **1a** and **1b** exhibited reduced membrane interactions and low toxicity in cell-based assays. Specifically, confocal microscopy (Figure 6, top row) showed pronounced membrane association for Aβ_1-42_ aggregates (Aβ) formed in the absence of compounds, whereas those formed in the presence of **1a** and **1b** (Aβ-**1a**, Aβ-**1b**) appeared smaller and more dispersed along the membrane.

Accordingly, magnified images (Figure 6, middle row) revealed comparable co-localization of Aβ aggregates with GM1 across conditions, although aggregates formed in the presence of **1a** and **1b** displayed reduced size.

In agreement with these observations, sensitized-emission FRET analysis revealed a strong signal in cells exposed to Aβ_1-42_ aggregates formed in the absence of compounds, indicating close-range interactions between cell-associated Aβ aggregates and GM1-enriched membrane domains. In contrast, aggregates generated in the presence of compounds **1a** and **1b** exhibited markedly reduced FRET signals (Figure 6, bottom row), suggesting a decreased ability to establish close membrane contacts. Notably, although some membrane-associated fluorescence could still be observed, the reduced FRET signal indicates that these aggregates were less frequently located within the nanometer-scale distances required for energy transfer. Together, these findings support the conclusion that compounds **1a** and **1b** alter the aggregation pathway of Aβ_1-42_, leading to the formation of structurally distinct aggregates with a reduced propensity to engage membrane GM1 domains under the experimental conditions tested.

#### 3.5.3. Reduced Cytotoxicity of Aβ_1-42_ Aggregates Generated in the Presence of Compounds **1a** and **1b**

**Figure 7:**
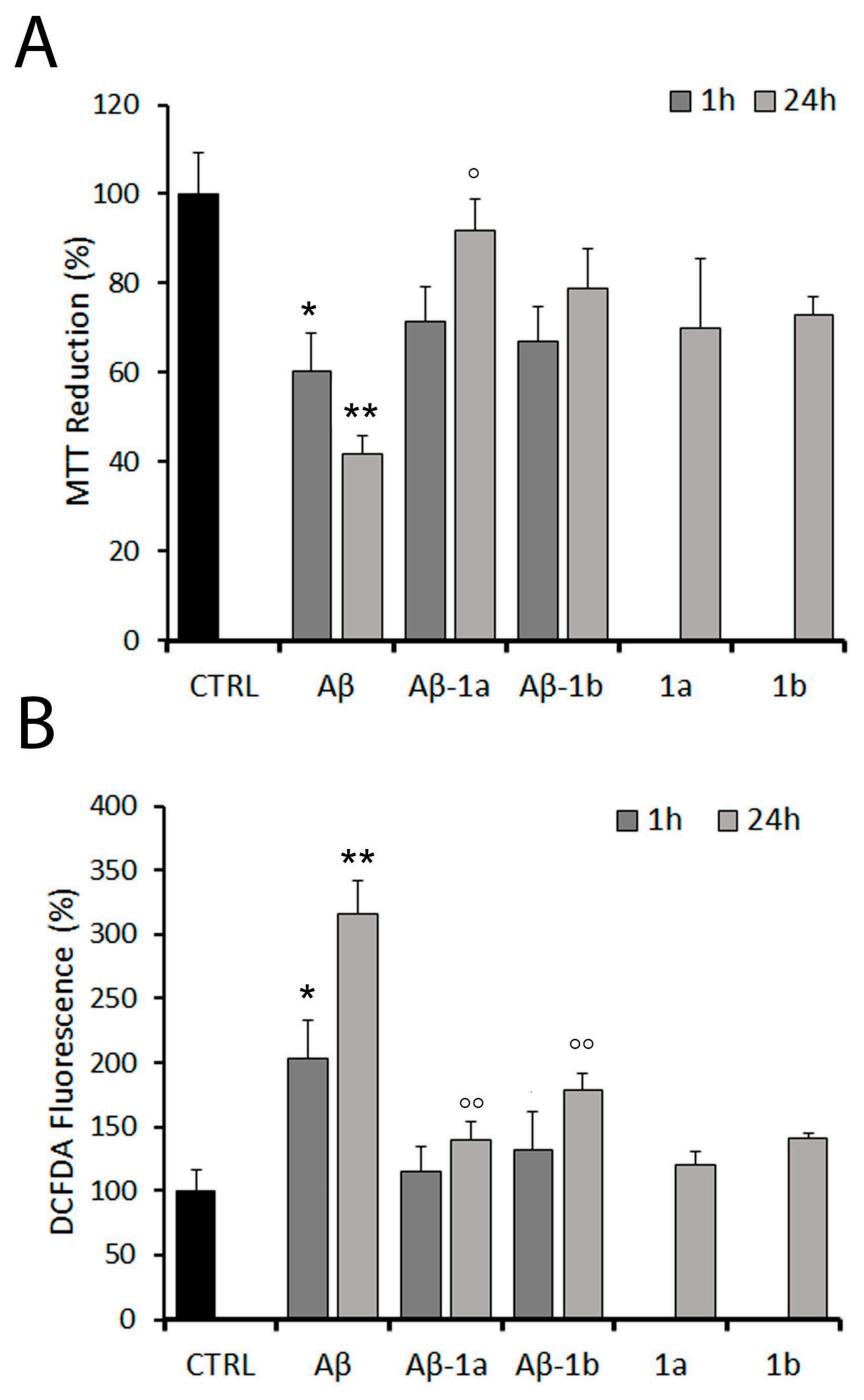
Reduced toxicity of Aβ_1-42_ aggregates generated in the presence of compounds **1a** and **1b**. RA-SH-SY5Y cells were exposed for 24 h to Aβ aggregates generated after 1 h or 24 h of incubation, either in the absence or presence of compounds **1a** and **1b** (2.5 μM, monomer equivalent). CTRL indicates untreated cells, whereas **1a** and **1b** indicate cells exposed to compounds alone (1 μM). (**A**) Cell viability was assessed by the MTT reduction assay. (**B**) Intracellular ROS levels were quantified using the CM-H_2_DCFDA fluorescent probe. Data are presented as mean ± SE from three independent experiments, each performed in triplicate. Statistical analyses were performed using one-way ANOVA followed by Tukey’s multiple-comparison test for comparisons with CTRL and two-way ANOVA followed by Tukey’s multiple-comparison test to evaluate the effects of aggregation time, treatment, and their interaction. Significant treatment effects were observed for both cell viability (F(2,48) = 3.63, *p* = 0.034) and ROS production (F(2,48) = 35.20, *p* < 0.0001). * *p* < 0.05, ** *p* < 0.01 vs. CTRL; ° *p* < 0.05, °° *p* < 0.01 vs. the corresponding untreated Aβ group.

The effects of Aβ_1-42_ aggregates generated in the absence or presence of compounds **1a** and **1b** were further evaluated in RA-SH-SY5Y cells using the MTT assay (Figure 7), which assesses cell viability based on the ability of metabolically active cells to reduce the tetrazolium salt MTT into insoluble formazan crystals.

Importantly, the intrinsic cytotoxicity of compounds **1a** and **1b** was assessed over a concentration range of 2.5 nM to 25 μM, and no significant reduction in cell viability was observed (Figure S4), indicating that the compounds are not inherently toxic under the experimental conditions used.

Exposure to Aβ_1-42_ aggregates significantly reduced cell viability compared with untreated controls, as confirmed by one-way ANOVA (F(2,24) = 7.69, *p* = 0.0026). In contrast, aggregates generated in the presence of compounds **1a** and **1b** showed reduced cytotoxicity, indicating that these compounds attenuate the deleterious effects associated with Aβ aggregation. Consistent with this observation, two-way ANOVA revealed a significant effect of treatment on cell viability (F(2,48) = 3.63, *p* = 0.034), whereas neither aggregation time nor the treatment × aggregation time interaction was significant. The strongest protective effect was observed for compound **1a**, for which Tukey’s post hoc analysis revealed a significant reduction in toxicity after 24 h of aggregation (*p* = 0.013). Under these conditions, cell viability approached levels comparable to untreated controls (up to ~95%).

To further explore the functional consequences of these different aggregates, intracellular ROS levels were measured in RA-SH-SY5Y cells exposed to Aβ_1-42_ aggregates formed in the absence or presence of **1a** and **1b** (Figure 7B). One-way ANOVA confirmed a significant increase in ROS production following exposure to Aβ aggregates (F(2,24) = 41.87, *p* < 0.0001). Two-way ANOVA revealed significant effects of aggregation time (F(1,48) = 23.96, *p* < 0.0001), treatment (F(2,48) = 35.20, *p* < 0.0001), and their interaction (F(2,48) = 8.69, *p* = 0.00061). Tukey’s multiple-comparison test showed significantly lower ROS levels in cells exposed to Aβ aggregates generated in the presence of compounds **1a** and **1b** after 24 h of aggregation compared with the corresponding untreated Aβ aggregates.

As expected, aggregates formed in the absence of compounds triggered a marked increase in ROS production, which became more pronounced after 24 h of aggregation. In contrast, aggregates generated in the presence of compounds **1a** and **1b** elicited a markedly attenuated oxidative response, indicating that these compounds reduce the ability of Aβ aggregates to promote oxidative stress. The reduction in oxidative stress paralleled the improved cell viability observed in the MTT assay, further supporting the notion that aggregates formed in the presence of compounds **1a** and **1b** possess reduced neurotoxic potential.

Collectively, these findings support a model in which compounds **1a** and **1b** redirect Aβ_1-42_ aggregation toward structurally distinct, less membrane-active species, thereby attenuating downstream toxic effects, including oxidative stress and loss of cell viability.

## 4. Discussion and Conclusions

Aβ_1-42_ aggregation is one of the most recognized hallmarks of AD and follows a well-characterized multistep pathway in which soluble monomers assemble into prefibrillar oligomers, which subsequently evolve through fibrillar intermediates to form mature Aβ fibrils [45]. Conventional therapeutic approaches have largely focused on inhibiting aggregation or promoting disaggregation, aiming to reduce Aβ_1-42_-mediated neurotoxicity [46,47].

However, increasing evidence indicates that Aβ_1-42_ aggregation is not a linear process leading exclusively to toxic species. Soluble oligomers can follow alternative assembly pathways, giving rise to structurally distinct, non-toxic species, while fibrillar intermediates and mature fibrils can further associate into high-molecular-weight, disordered aggregates, often referred to as off-pathway macroaggregates. Some of these higher-order assemblies have been reported to display lower toxicity than soluble oligomers and have therefore been proposed to sequester potentially harmful intermediates. In this context, an alternative therapeutic strategy has emerged, based on promoting the conversion of toxic Aβ species into less harmful aggregated states through pro-aggregating agents. Although still relatively unexplored, this approach has been reported for a limited number of compounds, particularly among polyphenolic and natural products.

In the present study, we investigated a series of polyphenolic imidazo[1,2-*a*]pyridines derived from the in-house compound **1a**, previously reported as a SIRT1 activator, as multifunctional compounds for future investigation in the context of AD.

On this basis, **1a** and a small set of closely related analogues, including a constitutional isomer and the corresponding methoxy derivatives, were synthesized through an improved synthetic pathway and subsequently characterized in vitro for their biological properties, including antioxidant and metal-ion chelating activities, as well as their impact on Aβ_1-42_ aggregation and toxicity.

The antioxidant profile of the compounds, assessed by DPPH, ABTS, and TBARS assays, highlighted a clear structure–activity relationship, with phenolic derivatives, **1a** and **1b**, showing markedly higher radical-scavenging and lipid peroxidation-inhibiting activity compared to their methoxy analogues, **2a** and **2b**. These results underscore the crucial role of free phenolic groups, particularly the catechol moiety, in stabilizing radical species and protecting against oxidative damage. Specifically, TBARS results demonstrate protection against chemically induced lipid peroxidation under the assay conditions. In parallel, UV–Vis studies indicated that selected derivatives interact with redox-active metal ions implicated in AD pathology. In particular, **1a** and **2a** showed a pronounced interaction with Cu^2+^, forming 2:1 metal-to-ligand complexes, while **1b** displayed a weaker interaction with Fe^2+^. Although further studies are required to fully characterize the nature of these complexes, these findings suggest that metal binding may complement the antioxidant activity of the compounds within their overall multifunctional profile.

The effects of these compounds on Aβ_1-42_ aggregation were then investigated using a combination of ThT fluorescence, TEM analysis, and intrinsic tyrosine fluorescence. The ThT assay revealed a marked reduction in fluorescence intensity in the presence of **1a** and **1b**. However, this decrease should not be interpreted as a simple inhibition of fibril formation. Indeed, TEM analysis revealed a greater abundance of fibrillar structures in the dried-grid preparations, suggesting that these compounds favor the formation and maturation of fibrillar Aβ assemblies.

This apparent discrepancy can be rationalized by considering that ThT fluorescence depends on the specific cross-β architecture and accessibility of binding sites within amyloid fibrils, rather than on fibril mass alone. Accordingly, the reduced ThT signal likely reflects the formation of structurally distinct fibrillar polymorphs displaying altered ThT-binding properties and/or aggregate architectures with reduced dye accessibility. Intrinsic tyrosine fluorescence measurements further supported this interpretation, showing a significant decrease in Tyr10 fluorescence intensity. Since the spectra were corrected by subtraction of the corresponding compound blanks, the observed quenching is unlikely to arise from direct spectral contributions of the compounds and is instead consistent with alterations in the local environment of Tyr10 accompanying the aggregation process.

Overall, these data suggest that, under the test conditions, compounds **1a** and **1b** do not inhibit Aβ_1-42_ aggregation but rather remodel the aggregation pathway, promoting the formation of structurally distinct fibrillar assemblies with altered ThT-binding properties. Importantly, this structural remodeling is associated with a marked reduction in the biological activity of Aβ_1-42_ assemblies.

Aggregates formed in the presence of **1a** and **1b** displayed markedly reduced FRET signals in GM1-enriched membrane domains, indicating a decreased propensity to establish the close-range membrane interactions observed for Aβ_1-42_ aggregates formed in the absence of the compounds. Consistently, these aggregates induced markedly lower cytotoxicity and ROS production in cell-based assays. These findings are consistent with the growing body of evidence indicating that soluble Aβ_1-42_ oligomers, rather than mature fibrils, are the primary mediators of neurotoxicity in AD, while highly ordered fibrils represent comparatively inert end-products [48,49].

In this context, the accelerated fibril formation observed in the presence of **1a** and **1b** is compatible with a possible redirection of the aggregation pathway toward structurally distinct and less toxic aggregated states.

Similar effects have been reported for a limited number of small molecules and polyphenols, including resveratrol, which can remodel Aβ_1-42_ aggregates into off-pathway species with reduced toxicity [46]. Additional examples include ZGM1, diacetyl, and betulinic acid, which promote aggregation toward less toxic states and improve functional outcomes in experimental models [47,50,51].

Moreover, our results align with recent studies indicating that small molecules can modulate Aβ_1-42_ aggregation kinetics and polymorphism without necessarily stabilizing toxic species [52]. In this framework, compounds **1a** and **1b** may shift the aggregation pathway toward less pathogenic end-states. The structural features of compound **1a**, particularly its polyphenolic hydroxylation pattern, may play a key role in this behavior, suggesting that specific substitution patterns could be exploited to design more effective aggregation-modulating agents.

Despite these encouraging results, the potential application of these compounds in the CNS/brain setting requires careful consideration of brain exposure. The structurally related imidazo[1,2-*a*]pyridine **GA11** showed activity following systemic administration in intracranial glioblastoma models, supporting the relevance of this chemotype in brain-related diseases [53,54]. However, SwissADME predictions [55] (https://www.swissadme.ch, accessed in September 2025) indicated a less favorable lipophilicity profile for the hydroxylated analogues **1a-b**, whereas the methoxy derivatives **2a-b** may have a greater propensity for passive BBB permeation. Thus, achieving adequate brain exposure may be challenging, particularly for the hydroxylated compounds, and future development may require alternative strategies, including intranasal nose-to-brain administration, [56,57] active targeting, [58] or formulation- and carrier-based delivery. [59,60] Notably, although resveratrol and its metabolites have been detected in cerebrospinal fluid, [61] its rapid metabolism and limited systemic bioavailability have prompted the development of several brain-targeted formulations, [62] thereby supporting the feasibility of alternative delivery approaches for compounds with limited passive BBB permeability.

In conclusion, compounds **1a** and **1b** combine antioxidant activity, metal-binding properties, and the ability to modulate Aβ_1-42_ aggregation within a single scaffold. Rather than inhibiting aggregation, they were associated, under the experimental conditions employed, with structurally distinct Aβ_1-42_ assemblies showing reduced membrane interaction, cytotoxicity, and ROS production. These findings provide preliminary support for further investigation of aggregation remodeling as a strategy to reduce the biological activity of Aβ_1-42_ assemblies.

Overall, the integration of antioxidant activity, metal binding, and aggregation remodeling within a single imidazo[1,2-*a*]pyridine scaffold highlights the potential of this chemotype as a potential multifunctional platform for lead optimization and subsequent mechanistic, in vivo validation in the context of AD.

## Figures and Tables

**Table 1:** Antioxidant activity of derivatives **1a-b** and **2a-b**, measured by the DPPH assay.

Cpd	Substitution Pattern	RSA% at 100 µM	EC_50_ (95% CI), µM
**1a**	3,5-diOH (4-OH)	22 ± 1	n.d.
**1b**	3,4-diOH (4-OH)	84 ± 0	49.2 (43.5–55.1)
**2a**	3,5-diOCH_3_ (4-OCH_3_)	36 ± 0	n.d.
**2b**	3,4-diOCH_3_ (4-OCH_3_)	21 ± 1	n.d.
Resveratrol			67.4 (64.9–69.9)

EC_50_ values are expressed in µM and reported as best-fit estimates with the corresponding 95% confidence intervals (CI) in parentheses. Values were obtained by nonlinear regression analysis using the “log(inhibitor) vs. normalized response—Variable slope” model in GraphPad Prism 9; CI values were calculated by profile likelihood. n.d.: not determined.

**Table 2:** Antioxidant activity of derivatives **1a-b** and **2a-b**, measured by the ABTS assay.

Cpd	Substitution Pattern	%RSA at 100 µM	EC_50_ (95% CI), µM
**1a**	3,5-diOH (4-OH)	78± 0	n.d.
**1b**	3,4-diOH (4-OH)	93 ± 0	13.7 (7–18)
**2a**	3,5-diOCH_3_ (4-OCH_3_)	20 ± 1	n.d.
**2b**	3,4-diOCH_3_ (4-OCH_3_)	38 ± 0	n.d.
Resveratrol			5.3 (3.5–7.9)

EC_50_ values are expressed in µM and reported as best-fit estimates with the corresponding 95% confidence intervals (CI) in parentheses. Values were obtained by nonlinear regression analysis using the “log(inhibitor) vs. normalized response—Variable slope” model in GraphPad Prism 9; CI values were calculated by profile likelihood. n.d.: not determined.

## Data Availability

The original contributions presented in this study are included in the article/Supplementary Material. Further inquiries can be directed to the corresponding authors.

## Supplementary Materials

The following supporting information can be downloaded at: https://www.mdpi.com/article/10.3390/antiox15070857/s1. Table of contents: NMR Characterization and HPLC Chromatograms of Final Compounds; TBARS Results; Chelating Activity Assays and MTT Assays of Compounds **1a** and **1b**.

## Abbreviations

The following abbreviations are used in this manuscript: ABTS, 2,2′-azino-bis(3-ethylbenzothiazoline-6-sulfonic acid); AD, Alzheimer’s disease; ADAM-10, A disintegrin and metalloproteinase 10; ANOVA, analysis of variance; Aβ, amyloid-β; Aβ_1-42_, amyloid-β_1-42_ peptide; BBB, blood–brain barrier; CM-H_2_DCFDA, 2′,7′-dichlorodihydrofluorescein diacetate; Cpd, compound; CTX-B, cholera toxin subunit B; DCF, 2′,7′-dichlorofluorescein; DCM, dichloromethane; DMEM, Dulbecco’s modified Eagle medium; DMSO, dimethyl sulfoxide; DPPH, 2,2-diphenyl-1-picrylhydrazyl; EC_50_, half maximal effective concentration; FBS, fetal bovine serum; FRET, Förster Resonance Energy Transfer; GM1, ganglioside GM1; HAM/F12, Ham’s F12 medium; MDA, malondialdehyde; MTT, 3-(4,5-dimethylthiazol-2-yl)-2,5-diphenyltetrazolium bromide; NAD^+^, nicotinamide adenine dinucleotide; NMR, nuclear magnetic resonance; NFT, neurofibrillary tangle; n.d., not determined; NMDA, N-methyl-D-aspartate; PBS, phosphate-buffered saline; RA, retinoic acid; RA-SH-SY5Y, retinoic acid-differentiated SH-SY5Y cells; ROS, reactive oxygen species; RSA, radical scavenging activity; RSA%, percentage radical scavenging activity; SD, standard deviation; SE, standard error; SH-SY5Y, human neuroblastoma cell line; SIRT1, sirtuin 1; SIRT6, sirtuin 6; TBARS, thiobarbituric acid reactive substances; TEM, transmission electron microscopy; ThT, thioflavin T; TLC, thin-layer chromatography; UV-Vis, ultraviolet–visible spectroscopy.
