# Supplementary material for: Polyphenolic Imidazopyridines as Multifunctional Modulators of Oxidative Stress, Metal Dyshomeostasis, and β1-42 Amyloid Aggregation in an In Vitro Model of Alzheimer’s Disease
Source: Antioxidants (Basel). 2026 Jul 8;15(7):857. doi: 10.3390/antiox15070857 (PMC13404437; doi:10.3390/antiox15070857)
Supplement: Supplementary file 1 [file antioxidants-15-00857-s001.zip › antioxidants-4364312-supplementary.pdf]

Supporting Information for Article

Polyphenolic Imidazopyridines as Multifunctional Modulators of Oxidative Stress, Metal Dyshomeostasis, and β1-42 Amyloid Aggregation in an In Vitro Model of Alzheimer's Disease

Lidia Ciccone [1,*], Giovanni Petrarolo [1], Ilaria D'Agostino [1,*], Fabio Scianò [1], Bianca Laura Bernardoni [1], Manuela Leri [2], Jihyae Ann [3], Susanna Nencetti [1], Jeewoo Lee [3], Monica Bucciantini [2] and Concettina La Motta [1]

[1] Department of Pharmacy, University of Pisa, 56126 Pisa, Italy; giovanni.petrarolo@farm.unipi.it (G.P.);
fabio.sciano@farm.unipi.it (F.S.); bianca.bernardoni@farm.unipi.it (B.L.B.);
susanna.nencetti@unipi.it (S.N.); concettina.lamotta@unipi.it (C.L.M.)

[2] Department of Experimental and Clinical Biomedical Sciences "Mario Serio", University of Florence,
50134 Florence, Italy; manuela.leri@unifi.it (M.L.); monica.bucciantini@unifi.it (M.B.)

[3] College of Pharmacy, Seoul National University, Seoul 08826, Republic of Korea;
jihuya@snu.ac.kr (J.A.); jeewoo@snu.ac.kr (J.L.)

* Correspondence: ilaria.dagostino@unipi.it (I.D.A.), lidia.ciccone@unipi.it (L.C.);

Table of Contents

NMR Characterization and HPLC Chromatograms of Final Compounds ---------------------------- S2

TBARS Results-- S8

Chelating Activity Assays-- S9

MTT Assays of Compounds 1a and 1b -- S11

NMR Characterization and HPLC Chromatograms of Final Compounds

5-(2-(4-Hydroxyphenyl)imidazo[1,2-*a*]pyridin-6-yl)benzene-1,3-diol (1a)

^{1}H NMR (400 MHz, DMSO-d_6) spectrum

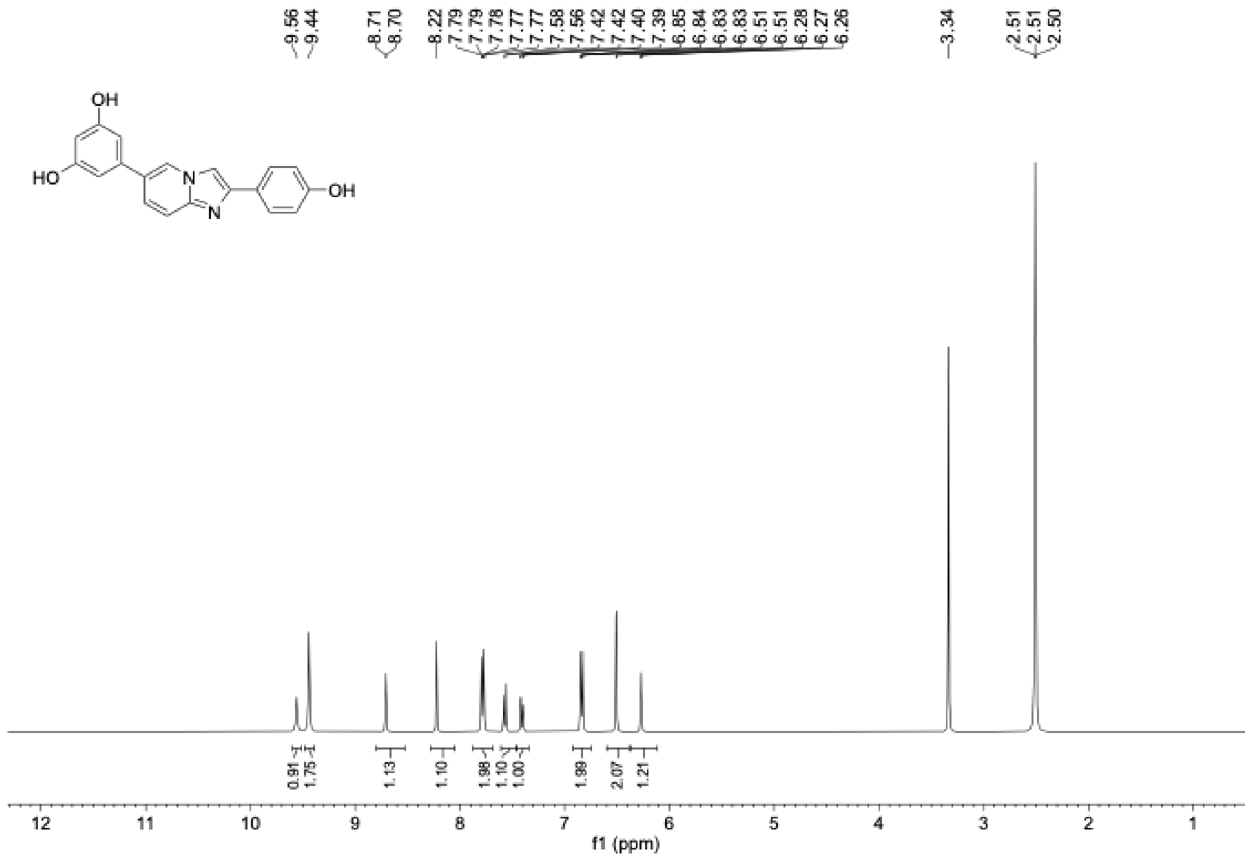

HPLC chromatogram (250-258 nm)

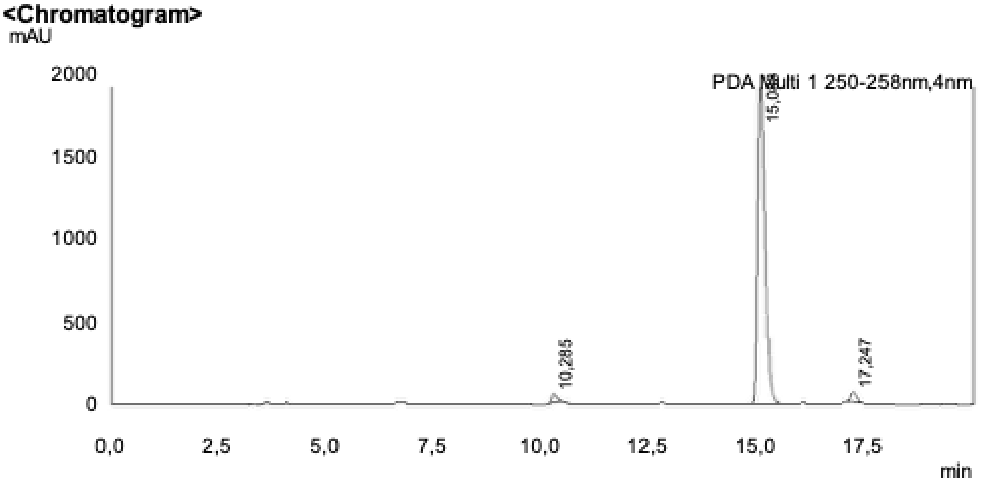

<Peak Table>

PDA Ch1 250-258nm

Peak#	Ret. Time	Height	Area	Height%	Area%
1	10.285	55715	463628	2.743	1.865
2	15.083	1914696	23792207	94.282	95.724
3	17.247	60398	599180	2.974	2.411
Total		2030810	24855015	100.000	100.000

4-(2-(4-Hydroxyphenyl)imidazo[1,2-*a*]pyridin-6-yl)benzene-1,2-diol (1b)

^{1}H NMR (400 MHz, DMSO-*d*$_6$) spectrum

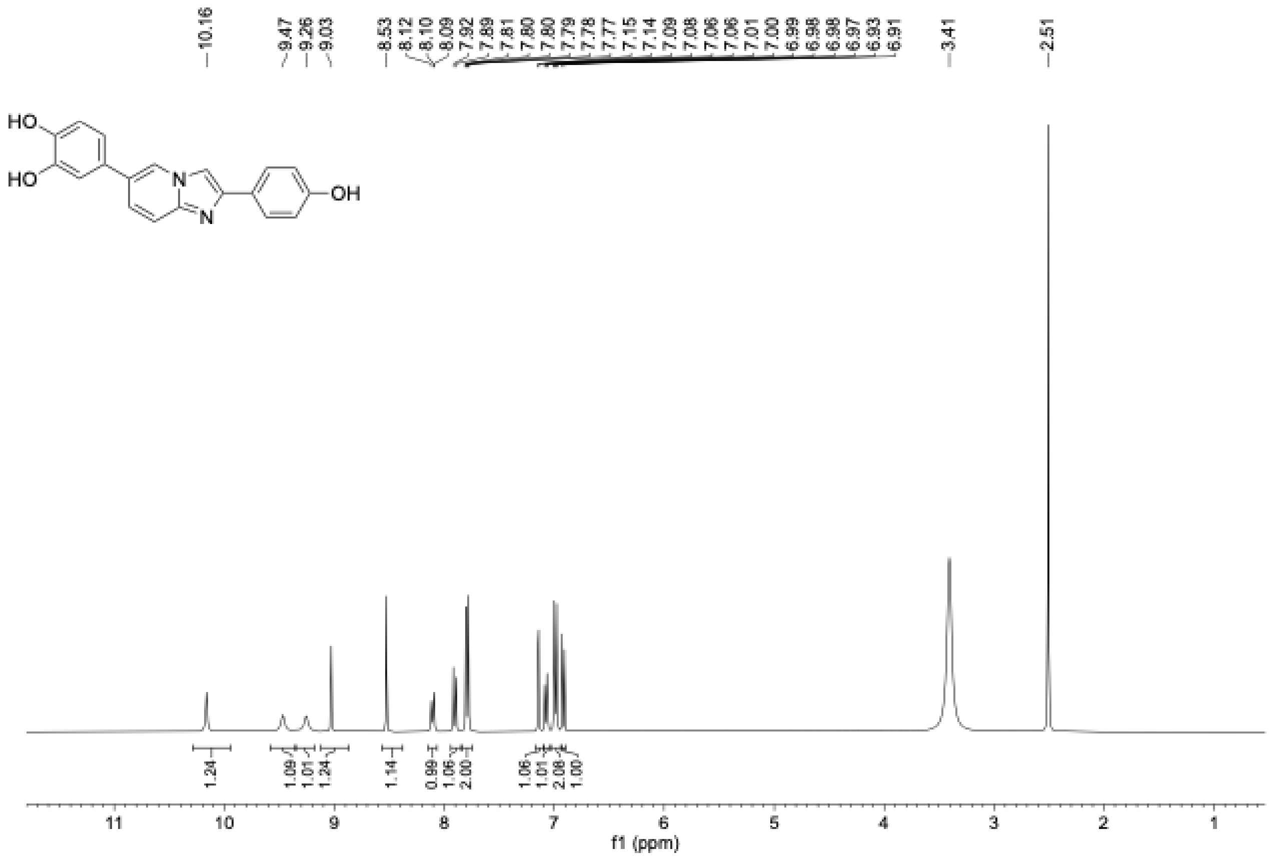

HPLC chromatogram (250-258 nm)

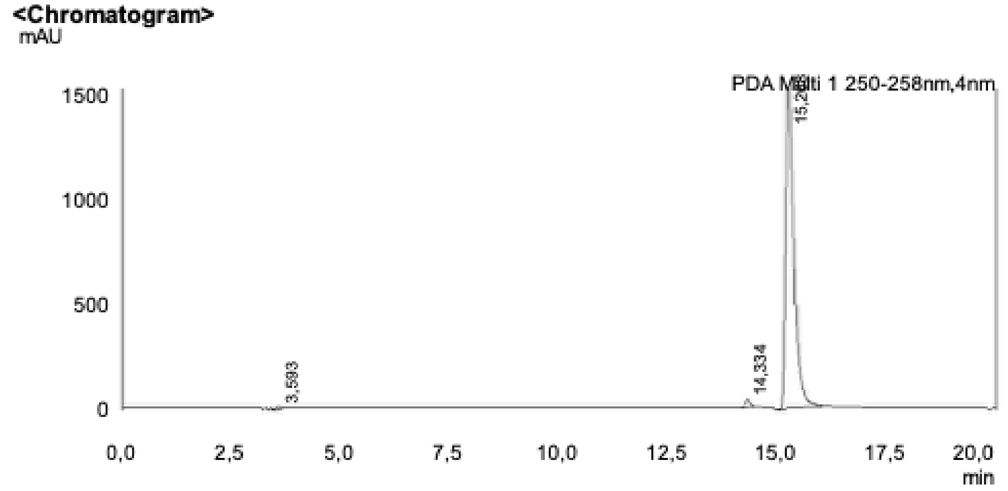

<Peak Table>

PDA Ch1 250-258nm

Peak#	Ret. Time	Height	Area	Height%	Area%
1	3.593	13510	121255	0.851	0.548
2	14.334	38043	316912	2.396	1.433
3	15.268	1535912	21673011	96.752	98.018
Total		1587465	22111178	100.000	100.000

6-(3,5-Dimethoxyphenyl)-2-(4-methoxyphenyl)imidazo[1,2-*a*]pyridine (2a)

^{1}H NMR (400 MHz, DMSO-d_6) spectrum

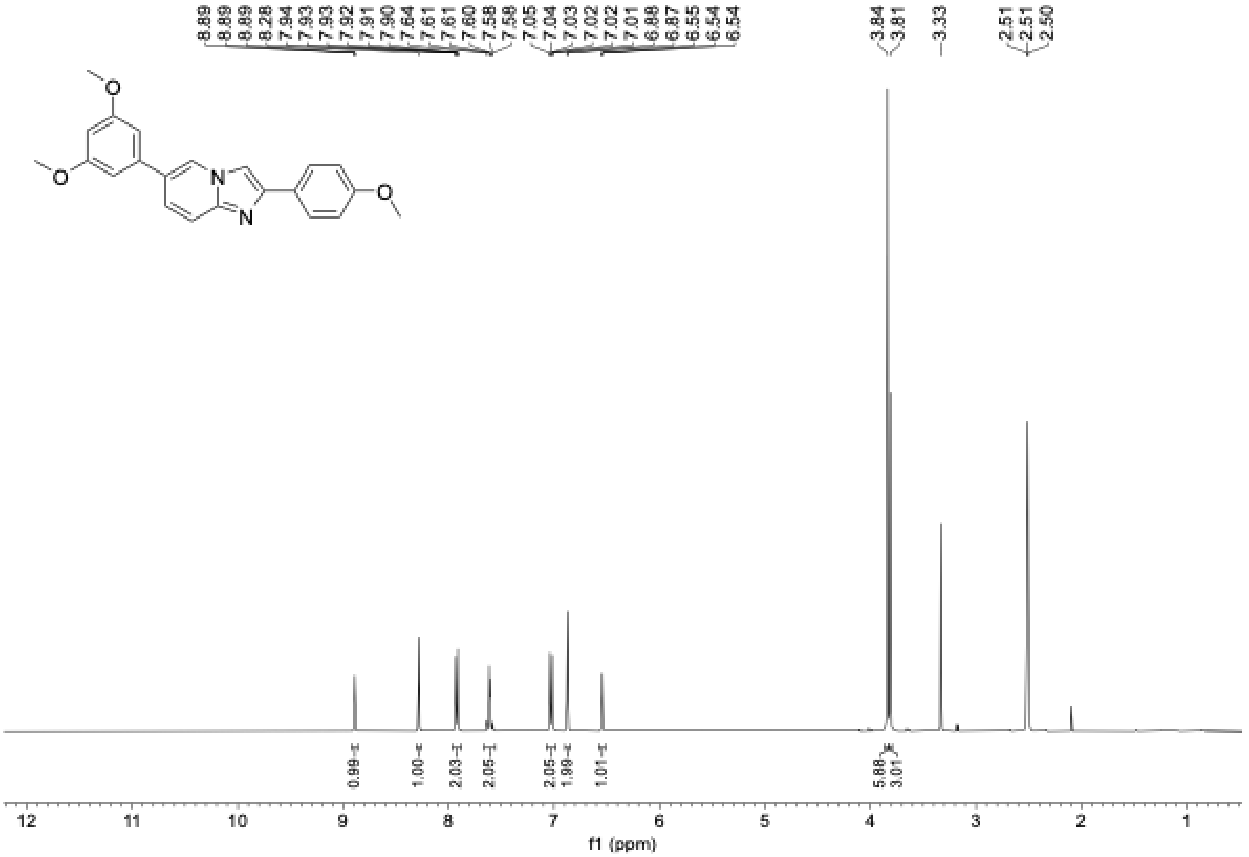

HPLC chromatogram (250-258 nm)

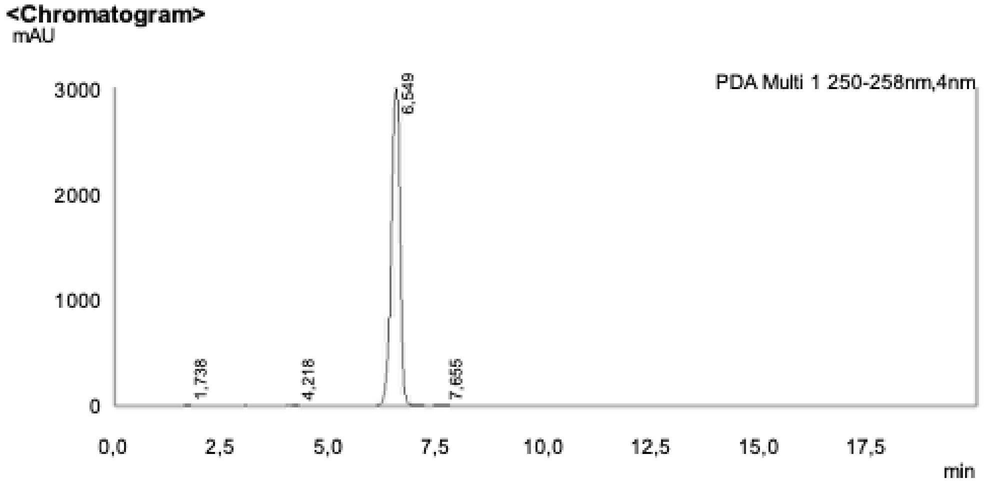

<Peak Table>

PDA Ch1 250-258nm

Peak#	Ret. Time	Height	Area	Height%	Area%
1	1.738	12183	101895	0.397	0.237
2	4.218	14901	113587	0.486	0.265
3	6.549	3030968	42436267	98.774	98.872
4	7.655	10547	268679	0.344	0.626
Total		3068599	42920427	100.000	100.000

6-(3,4-Dimethoxyphenyl)-2-(4-methoxyphenyl)imidazo[1,2-*a*]pyridine (2b)

^{1}H NMR (400 MHz, DMSO-*d*$_6$) spectrum

.

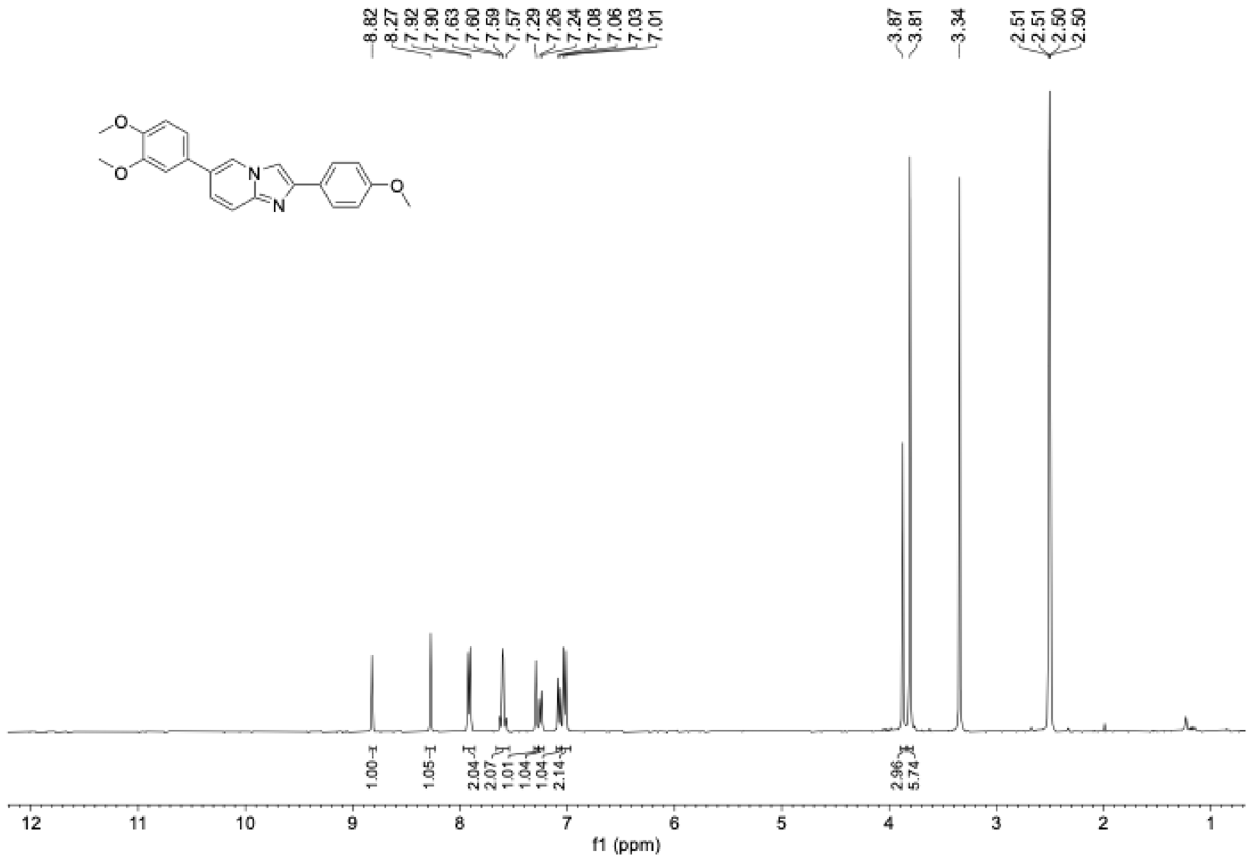

HPLC chromatogram (250-258 nm)

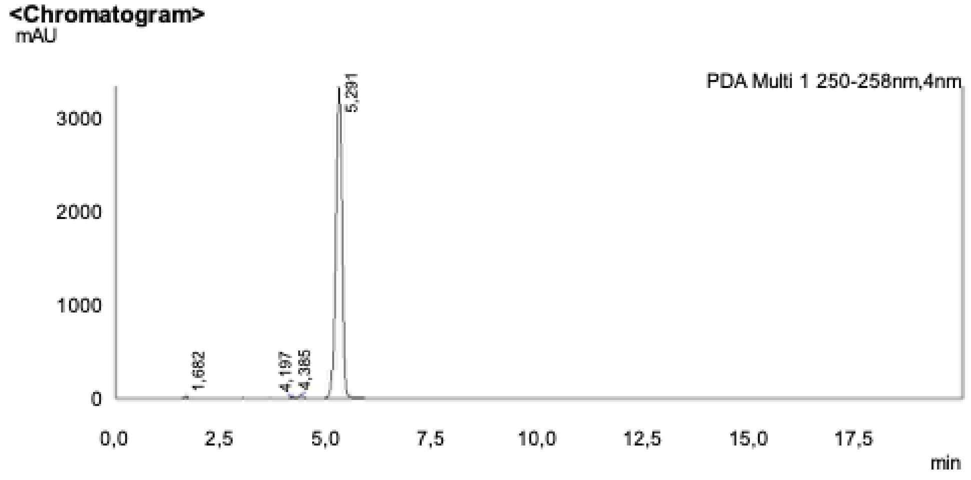

<Peak Table>

PDA Ch1 250-258nm

Peak#	Ret. Time	Height	Area	Height%	Area%
1	1.682	23167	139559	0.676	0.388
2	4.197	20212	161266	0.590	0.449
3	4.385	38094	316542	1.112	0.881
4	5.291	3345132	35327373	97.622	98.282
Total		3426606	35944740	100.000	100.000

5-(3,5-Dimethoxyphenyl)pyridin-2-amine (3a)

^{1}H NMR (400 MHz, DMSO-d_6) spectrum

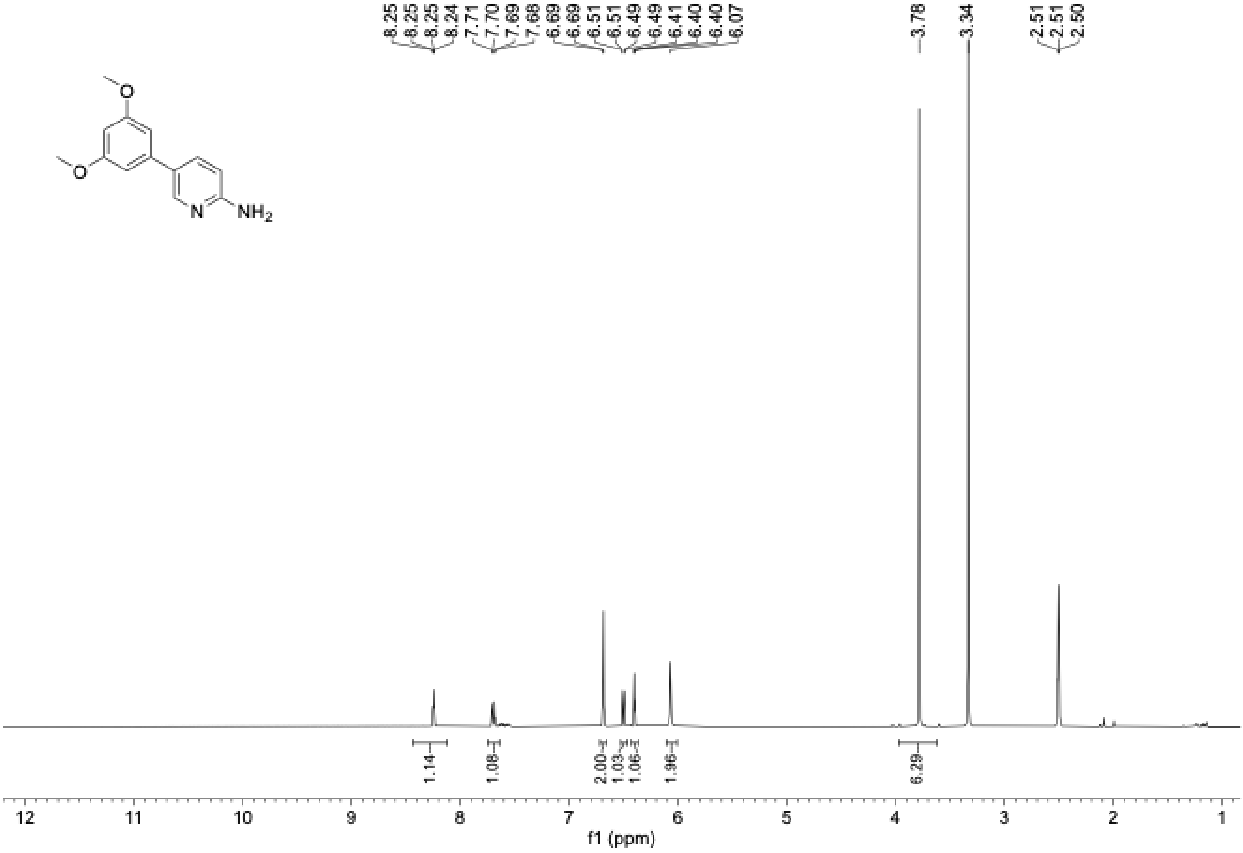

5-(3,4-Dimethoxyphenyl)pyridin-2-amine (3b)

^{1}H NMR (400 MHz, DMSO-d_6) spectrum

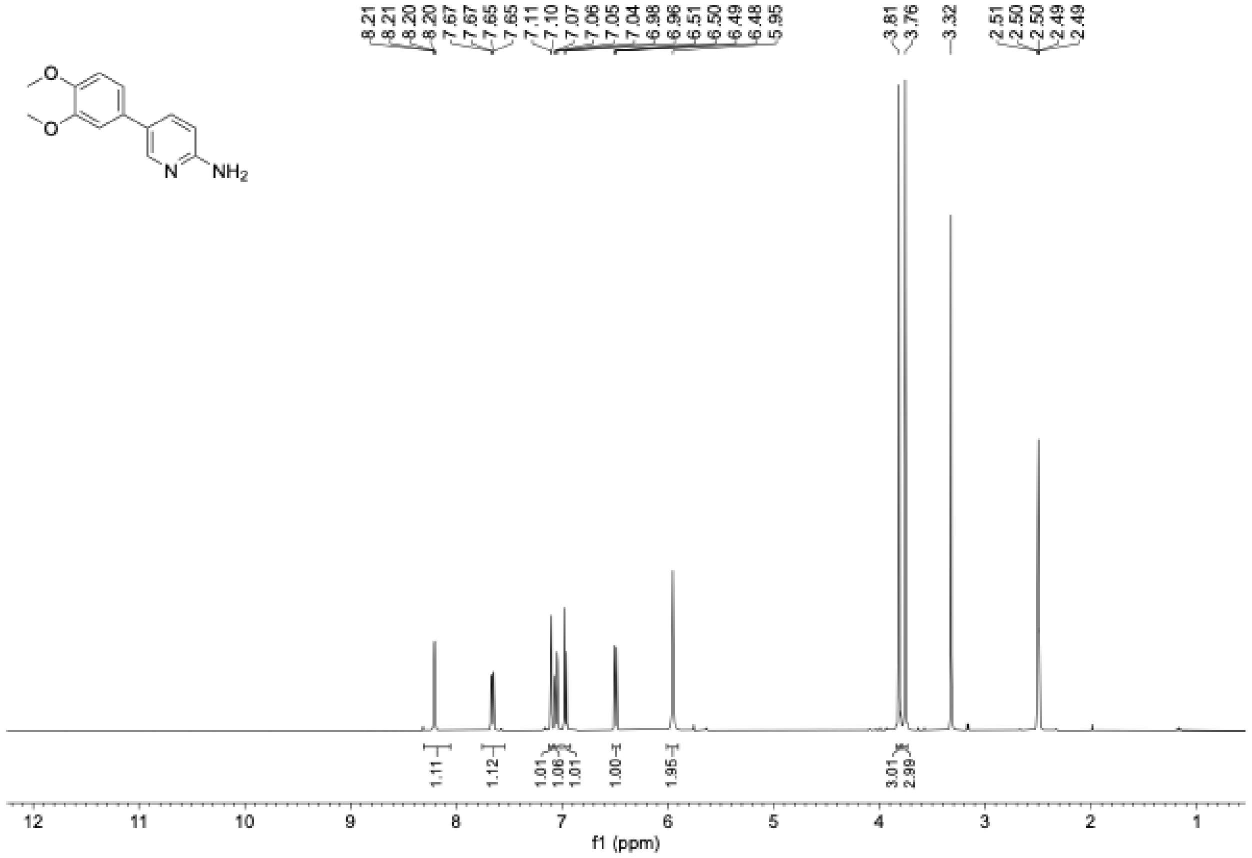

TBARS Results

Table S1: TBARS results, expressed as nmol of MDA per 10 mg of rat brain.

Sample	Tested concentration (μM)					
	100		10		1	
	nmol MDA ± SD	% Oxidation inhibition	nmol MDA ± SD	% Oxidation inhibition	nmol MDA ± SD	% Oxidation inhibition
**Control**	7.81±0.20	0				
**Basal**	2.20±0.10	72				
**Resveratrol**	2.43±0.04	69	5.70±0.47	30	8.18±0.35	0
**1a**	1.23±0.11	84	4.05±0.19	48	7.46±0.45	5
**1b**	1.20±0.15	85	1.20±0.05	85	4.15±0.38	47
**2a**	3.70±0.26	53	6.33±0.15	19	8.26±0.21	0
**2b**	1.26±0.02	84	2.48±0.46	8	4.59±0.42	42

Values are reported as nmol MDA ± standard deviation (SD) of three independent experiments and relative percentage (%) of oxidation inhibition.

Chelating Activity Assays

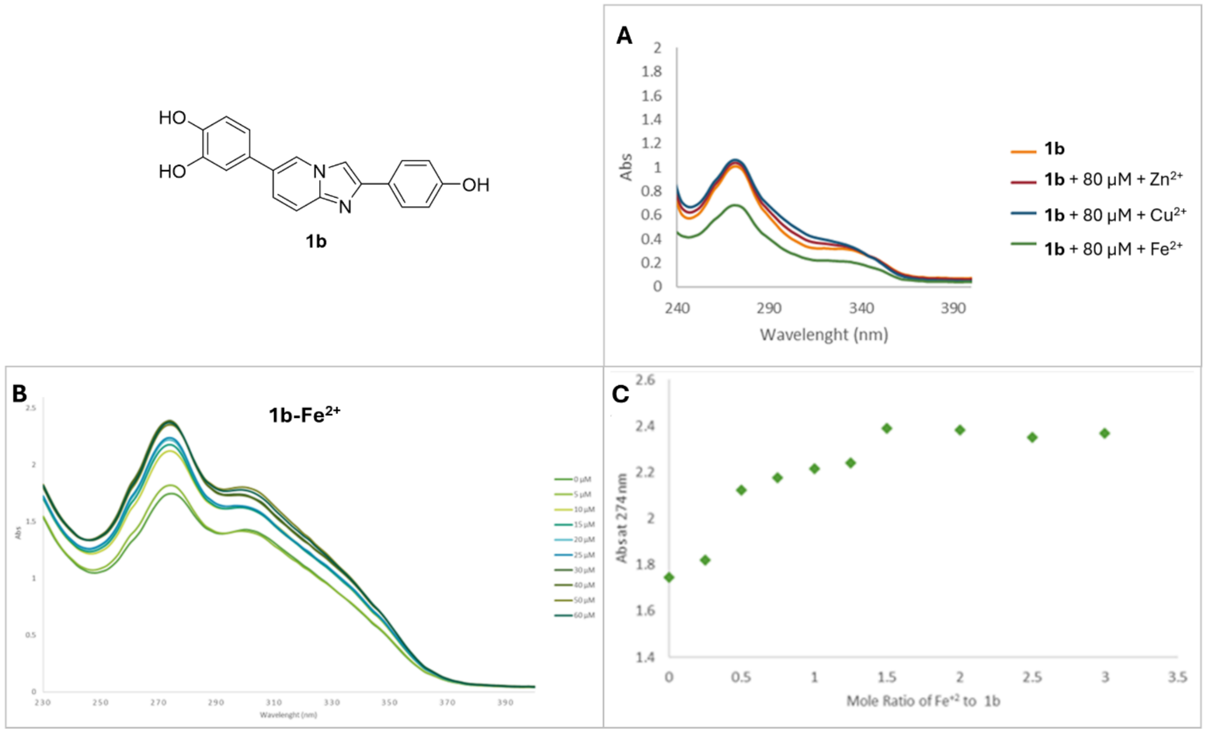

Figure S1: A) UV spectra of **1b** (20 μM) in the presence of Zn^{2+}, Cu^{2+}, and Fe^{2+} (80 μM); **B**) UV spectra of **1b** (20 μM) in the presence of different concentrations of Fe^{2+} (0-80 μM); **C**) determination of the stoichiometry of the **1b**-Fe^{2+} complex.

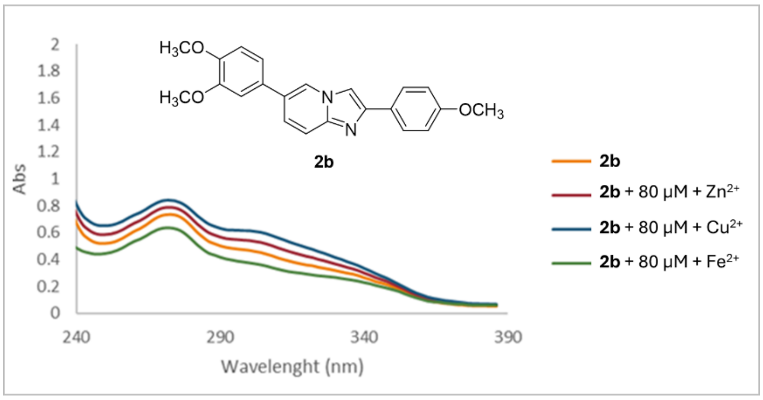

Figure S2: UV spectra of compound **2b** (20 μM) in the presence of Zn^{2+}, Cu^{2+}, and Fe^{2+} (80 μM).

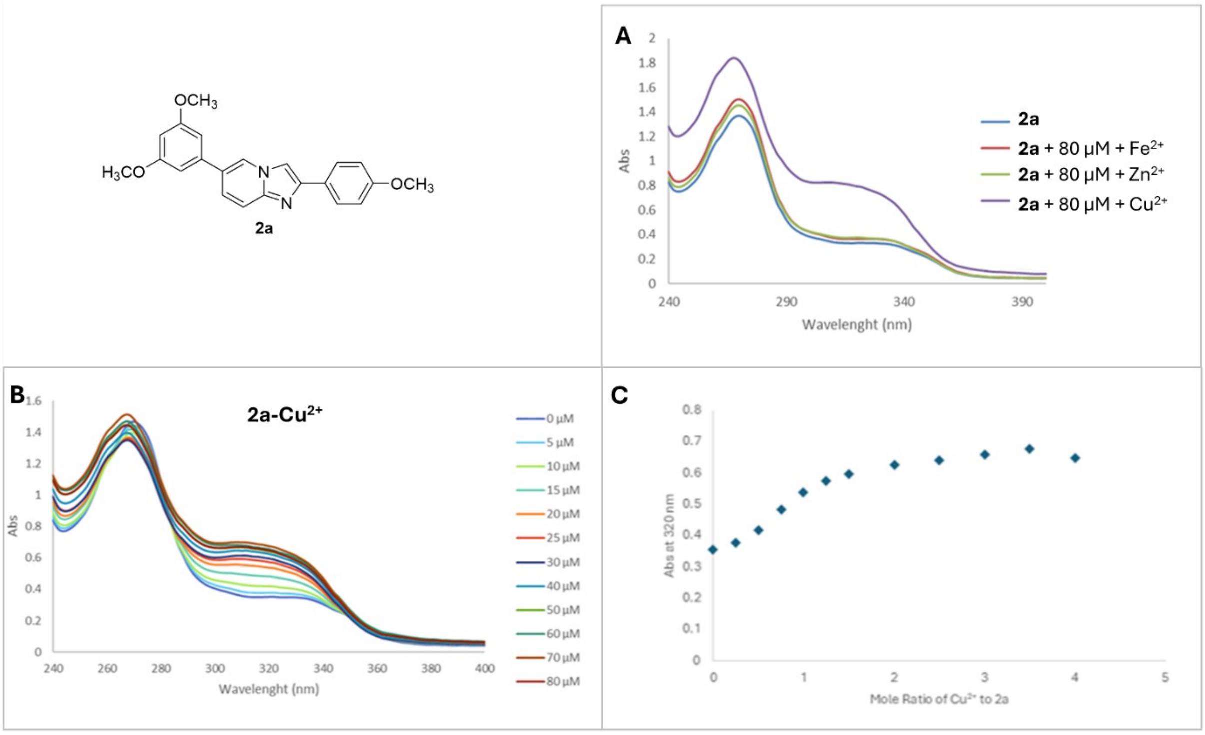

Figure S3: A) UV spectra of **2a** (20 μM) in the presence of Zn^{2+}, Cu^{2+}, and Fe^{2+} (80 μM); **B**) UV spectra of **2a** (20 μM) in the presence of different concentrations of Fe^{2+} (0-80 μM); **C**) determination of the stoichiometry of the **2a**-Cu^{2+} complex.

MTT Assays of Compounds 1a and 1b

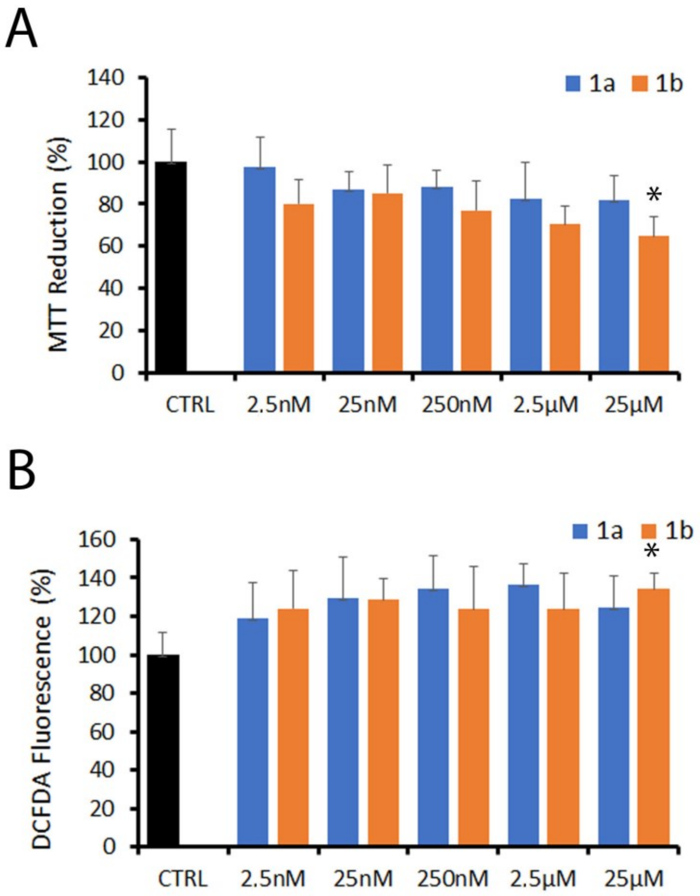

Figure S4: RA-SH-SY5Y cells were exposed for 24 h to different concentrations of compounds **1a** and **1b. A**) Cell Viability was assessed by measuring the MTT reduction assay; **B**) Intracellular ROS levels were quantified using the CM-H_2-DCFDA fluorescent probe. Data are presented as the mean ± standard error (SE) from three independent experiments, each performed in triplicate. Statistical analysis: one-way ANOVA followed by Tukey's multiple-comparison test *p<0.05; vs CTRL (untreated cells).
